# Randomized double‐blind clinical studies of ularitide and other vasoactive substances in acute decompensated heart failure: a systematic review and meta‐analysis

**DOI:** 10.1002/ehf2.12349

**Published:** 2018-09-24

**Authors:** Veselin Mitrovic, Wolf‐Georg Forssmann, Jan Schnitker, Stephan B. Felix

**Affiliations:** ^1^ Kerckhoff‐Klinik Forschungsgesellschaft mbH Küchlerstrasse 10 61231 Bad Nauheim Germany; ^2^ Department of Internal Medicine, Clinic of Immunology, Division of Peptide Research Hannover Medical School (MHH) Hannover Germany; ^3^ Institute of Applied Statistics (IAS) Ltd Bielefeld Germany; ^4^ Department of Internal Medicine B University Medicine Greifswald and DZHK (German Centre for Cardiovascular Research), partner site Greifswald Greifswald Germany

**Keywords:** Acute decompensated heart failure, Meta‐analysis, Placebo, Systematic review, Ularitide, Vasoactive substances

## Abstract

**Aims:**

Acute decompensated heart failure (ADHF) has a poor prognosis and limited treatment options. No direct comparisons between ularitide—a synthetic natriuretic peptide being evaluated in ADHF—and other vasoactive substances are available. The aim of this meta‐analysis was to determine haemodynamic effect sizes from randomized double‐blind trials in ADHF.

**Methods and results:**

Eligible studies enrolled patients with ADHF requiring hospitalization and haemodynamic monitoring. Patients received 24–48 h of infusion with a vasoactive substance or comparator. Primary outcome measure was pulmonary artery wedge pressure (PAWP). Treatment effects were quantified as changes from baseline using mean differences between study drug and comparator. Results were analysed using random‐effects (primary analysis) and fixed‐effects meta‐analyses. Twelve randomized, double‐blind studies were identified with data after 3, 6, and 24 h of treatment (*n* = 622, 644, and 644, respectively). At 6 h, significant PAWP benefits for ularitide over placebo were seen (Hedges' *g* effect size, −0.979; *P* < 0.0001). On meta‐analysis, treatment difference between ularitide and pooled other agents was statistically significant (−0.501; *P* = 0.0303). Effect sizes were numerically higher with ularitide than other treatments at 3 and 24 h. After 6 h, a significant difference in effect size between ularitide and all other treatments was observed for right atrial pressure (Hedges' *g*, −0.797 for ularitide and −0.304 for other treatments; *P* = 0.0274).

**Conclusions:**

After 6 h, ularitide demonstrated high effect sizes for PAWP and right atrial pressure. Improvements in these parameters were greater with ularitide vs. pooled data for other vasoactive drugs.

## Introduction

Acute decompensated heart failure (ADHF) is the most common form of acute heart failure, with an estimated annual incidence in the USA of 11.6 per 1000 persons aged 55 years or older.^2^ Inpatient treatment for ADHF has a poor prognosis, with a readmission rate of nearly 50% at 6 months and 1 year mortality of 30%.^3^ Management of ADHF focuses on decongestion and symptom improvement, with treatment of precipitating events and co‐morbid conditions.^3^ No randomized trial has demonstrated a beneficial effect of any drug on ADHF prognosis. Indeed, inotropic agents—particularly cyclic adenosine monophosphate‐generating drugs—increase myocardial oxygen consumption and induce arrhythmias^4^ and may also increase mortality.^5^ Similarly, long‐term oral phosphodiesterase III inhibitor therapy increases mortality in severe chronic heart failure.^6^

Recently, interest has focused on neurohormonal pathways underlying cardiac dysfunction and myocyte damage. Several drug classes have been developed to correct or mitigate disordered pathways and thereby preserve and protect myocytes. Natriuretic peptides are an endogenous family of structurally similar polypeptides that have vasodilatory, natriuretic, and antiproliferative effects on the heart.^7^ Ularitide is a synthetic form of urodilatin, a kidney‐derived natriuretic peptide involved in sodium and water homeostasis.^8^ Ularitide binds primarily to the extracellular domain of natriuretic peptide receptor A, which is expressed in the heart, the kidney, vascular smooth muscle tissue, and other organs. Efficacy and tolerability of ularitide were established in the Phase 2 SIRIUS studies^1^, ^9^; a Phase 3 study (Trial of Ularitide Efficacy and Safety in Acute Heart Failure) has been presented and published however with neutral effect on primary endpoints.^10^, ^11^

Positive haemodynamic effects have also been demonstrated in studies with other vasoactive substances including nesiritide, a second natriuretic peptide (the Natrecor Study Group)^12^; the calcium sensitizer levosimendan (the Levosimendan Infusion vs. Dobutamine (LIDO) study)^13^; the endothelin‐1 antagonist tezosentan (the Value of Endothelin Receptor Inhibition With Tezosentan in Acute Heart Failure Studies (VERITAS) studies)^14^; cinaciguat, an activator of soluble guanylate cyclase^15^; and serelaxin, a derivative of endogenous relaxin with vasodilator properties.^16^ Some studies suggested improvements in morbidity or mortality, supporting further clinical development, but had neither the duration nor the statistical power to assess these endpoints accurately. Longer‐term Phase 3 trials have not demonstrated reductions in mortality; thus, the role of vasoactive drugs in the management of ADHF remains uncertain. Furthermore, no head‐to‐head haemodynamic comparisons between vasoactive substances have yet been conducted in ADHF. Therefore, a systematic review was performed to demonstrate the evidence for treatment with ularitide in patients with ADHF.

### Hypothesis and purpose

This meta‐analysis was based on a pilot study including six studies—Safety and efficacy of an Intravenous placebo‐controlled Randomized Infusion of Ularitide in a prospective double‐blind Study (SIRIUS II),^1^ VMAC,^17^ LIDO,^13^ VERITAS (low‐dose tezosentan),^14^ a cinaciguat study,^15^ and a serelaxin study^16^ comparing effect sizes on pulmonary artery wedge pressure (PAWP) using unadjusted (naïve) and placebo‐adjusted data. Encouraging results led to a broader analysis that adhered to the Preferred Reporting Items for Systematic Reviews and Meta‐Analyses guidelines.^18^ The eligibility criteria for including randomized controlled trials and information sources, the outcomes to be analysed, and the study appraisal and synthesis methods were prospectively agreed by the authors in writing but not registered as a formal protocol.

The objective was to determine effect sizes from randomized double‐blind haemodynamic studies that included 24–48 h of treatment with ularitide or other vasoactive substances vs. placebo or active comparator for intravenous treatment of ADHF.

## Methods

This meta‐analysis has been reported in accordance with the Preferred Reporting Items for Systematic Reviews and Meta‐Analyses guidelines^18^ and the Cochrane Handbook.^19^ No published study protocol exists for this systematic review.

### Eligibility criteria

Eligible studies were conducted in patients with ADHF who required hospitalization and haemodynamic monitoring (right heart catheterization). All patients had lung congestion with elevated PAWP (variably defined as ≥15 to ≥20 mmHg) and were considered in need of acute intravenous therapy because of dyspnoea at rest or during minimal physical activity. All patients received an infusion with a vasoactive substance (or placebo/comparator) for 20–48 h plus standard therapy. Haemodynamic parameters were measured at baseline and after 2–4, 6–8, and 20–24 h. The primary endpoint was PAWP 6 h after starting infusion. Patients with chronic stable heart failure, for example, those in New York Heart Association Functional Class II, were excluded. Other exclusion criteria concerning the form or stage of heart failure, concomitant diseases, or history of significant illnesses, disallowed medication in the patient's history or at baseline, or disallowed conditions concerning the study conduct, were accepted as specified in individual studies.

### Information sources

The German Institute of Medical Documentation and Information interface was used to search the Cochrane Central Register of Controlled Trials and MEDLINE®; MEDLINE was also searched using PubMed with the Research Information Systems interface. The ClinicalTrials.gov database was searched using the rclinicaltrials package.^20^ This package provides a set of functions to interact with the search and download features of ClinicalTrials.gov. Results are downloaded to temporary directories and returned as R objects. Full search terms are listed in Supporting Information, *Table* *S1*.

### Study selection

The search shown in Supporting Information, *Table* *S1* resulted in 148 potentially eligible abstracts, which were independently checked for compliance with the eligibility criteria by J. S. and four colleagues; 114 abstracts could be excluded on the basis of 10 working criteria. The remaining 34 publications were included in the full‐text screening. The final selection was prepared by the group around J. S., but all decisions were made by the expert V. M.

From each study, we selected a study medication arm and placebo/active comparator arm. In studies with multiple treatment arms with different doses, the dose used according to the published Phase 3 studies was included. Data were collected from full‐text publications (text, tables, and figures as far as digitization was possible).

Haemodynamic outcomes presented are PAWP, cardiac index, right atrial pressure (RAP), systolic and diastolic blood pressure (SBP and DBP), and systemic vascular resistance (SVR). Pulmonary vascular resistance, mixed venous oxygen saturation, and transpulmonary pressure gradient were not systematically reported in the original publications and could not, therefore, be included in the meta‐analysis. Other outcomes of interest included serum creatinine, brain natriuretic peptide (BNP), and N‐terminal pro‐BNP (NT‐proBNP)—analysed as a cluster—and mortality rate after ~30 days.

Safety was evaluated in terms of rates of infusion discontinuation (total and due to adverse events) and rates of adverse events during infusion (total and serious).

### Statistical methods

Fixed‐effects and random‐effects models were used to estimate pooled effect sizes from aggregate data. Because heterogeneity was expected in the synthesis of studies with preparations other than ularitide, the use of the random‐effects model was pre‐specified for the primary analysis. Within single studies, treatment effects with respect to haemodynamic and specific laboratory parameters were quantified in terms of mean changes from baseline and transformed to standardized mean differences vs. comparator according to Hedges' *g*.^19^ Safety data were quantified by risk ratios for the study treatment vs. comparator.

The risk of bias in the individual studies was assessed using the Cochrane risk of bias tool.^19^ The comparison of effect sizes in different subgroups of studies, which is the main objective of meta‐analysis, is based on a partitioning of the complete variation of the effect sizes in the variance of the true effect (true heterogeneity) and partly spurious heterogeneity, incorporating both (true) heterogeneity and random error. The so‐called *I* ^2^ value gives the proportion of the (true) heterogeneity (in %) of the complete variation of the effect sizes. *I* ^2^ is calculated from Cochran's *Q* value by mean of *I* ^2^ = [*Q* − (*k* − 1)]∕*Q* with the number of studies *k*, and *Q* is the weighted sum of squared distances of the study means from the fixed effect estimated; weights are ‘inverse variance weights’.^21^, ^22^ The significance of *I* ^2^ was tested with the *Q* statistics. *I* ^2^ of approximately 50% was regarded as moderate and approximately 75% as considerable.

## Results

### Identified studies

**Figure 1:**
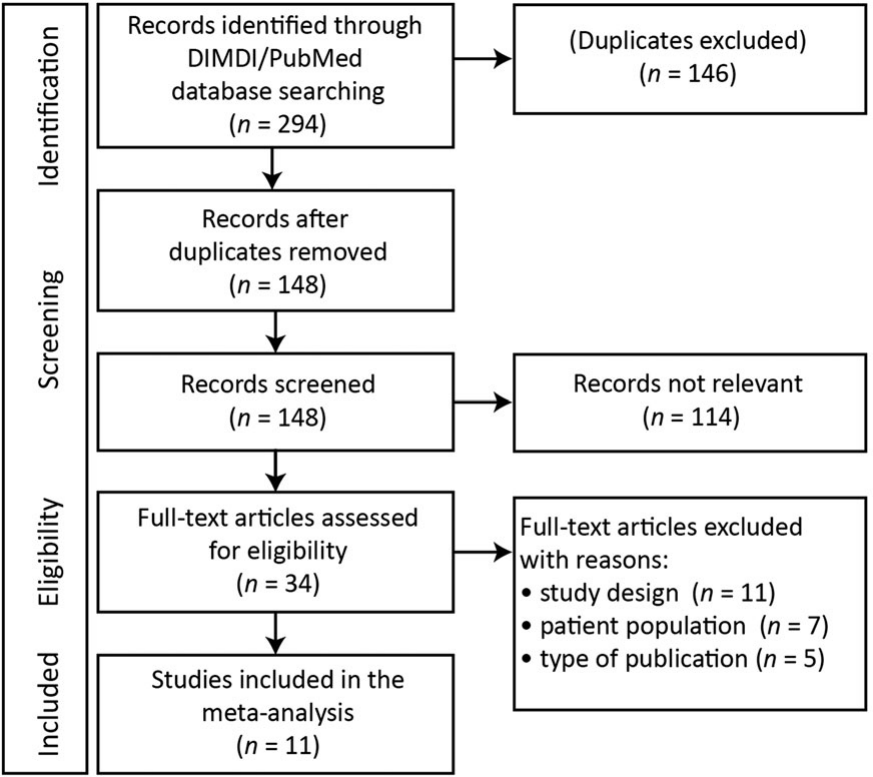
Preferred Reporting Items for Systematic Reviews and Meta‐Analyses flow diagram showing studies screened, eligible, and included in the meta‐analysis. DIMDI, Deutsches Institut für Medizinische Dokumentation und Information.

German Institute of Medical Documentation and PubMed searches identified 148 potentially eligible publications. No additional completed studies were identified by the ClinicalTrials.gov search. Screening of abstracts excluded all, but 34 publications were reduced to 11 after full‐text review (*Figure* *1*).^1^, ^9^, ^12^, ^13^, ^14^, ^15^, ^16^, ^17^, ^23^, ^24^, ^25^ Details of these studies are shown in *Table* 1. Available outcome measures per study and per time point after baseline (3, 6, and 24 h) are presented in Supporting Information, *Table* *S2*. All studies were double‐blind, randomized, controlled clinical trials vs. placebo (*n* = 10, including the first 3 h of the VMAC study^17^) and/or active comparator (*n* = 2, including the VMAC^17^ study from 3 h onwards; data with nitroglycerin therapy from 0 to 3 h in VMAC were ignored in favour of the placebo data). In addition to the nitroglycerin arm in VMAC,^17^ active comparators were used in LIDO (dobutamine 5 μg/kg/min),^13^ and Nieminen *et al*. (dobutamine 6 μg/kg/min).^24^ However, the dobutamine arm and an ethanol vehicle arm in the Nieminen *et al*. study were not included in the meta‐analysis, because these arms were not blinded.^24^ The Torre‐Amione *et al*. study^25^ used two doses of tezosentan (50 and 100 mg/h) that were substantially higher than the preferred dose of 1 mg/h in VERITAS.^14^ The 50 mg/h dose was selected after showing that both arms resulted in almost identical effect sizes for the main parameter (PAWP) after 6 h (data not shown).

**Table 1:** Characteristics of the 11 studies included in the meta‐analysis

Code	Study	Study design	Patient population	Study treatment (dose)		No. (selected dose/total)	Comparator	No.
ULAp	SIRIUS I^9^	DB RCT	ADHF (NYHA Class III/IV)	ULA (7.5, 15^a^, and 30 mg/kg/min)		6/18	Placebo	6
ULA	SIRIUS II^1^	DB RCT	ADHF, cardiac index ≤2.5 L/min/m^2^, and PAWP ≥18 mmHg	ULA (7.5, 15^a^, and 30 mg/kg/min)		53/168	Placebo	53
NESp	Mills *et al*.^12^	DB RCT	HF (NYHA Class II–IV), LVEF ≤35%, PAWP ≥18 mmHg, and cardiac index ≤2.7 L/min/m^2^	NES (0.015^a^, 0.03, and 0.06 μg/kg/min)		22/74	Placebo	29
NES	VMAC^17^	DB RCT, double‐dummy, placebo, and active comparator	Dyspnoea from decompensated CHF requiring hospitalization and intravenous therapy and PAWP ≥20 mmHg	NES (0.01 μg/kg/min)	≤3 h	124/124	Placebo^b^	62
					>3 h	146/146	Nitroglycerin^c^	85
LEVp	Nieminen *et al*.^24^	DB RCT	Stable CHF (NYHA Class III/IV) of ischaemic origin and LVEF <40%	LEV (0.05, 0.1^a^, 0.2, 0.4, and 0.6 μg/kg/min)		23/95	Placebo^d^	21
LEV	LIDO^13^	DB RCT, double‐dummy, and active comparator	Low‐output HF, LVEF <35%, cardiac index ≤2.5 L/min/m^2^, and PAWP >15 mmHg	LEV (0.1 μg/kg/min)		103/103	Dobutamine 5 μg/kg/min	100
TEZp	Cotter *et al*.^23^	DB RCT	ADHF, cardiac index <2.5 L/min/m^2^, and PAWP ≥20 mmHg	TEZ (0.2, 1^a^, 5, and 25 mg/h)		27/103	Placebo	26
TEZld	VERITAS^14^	DB RCT	ADHF, respiratory rate ≥24/min, cardiac index ≤2.5 L/min/m^2^, and PAWP ≥20 mmHg	TEZ (1 mg/h)		43/43	Placebo	41
TEZhd	Torre‐Amione *et al*.^25^	DB RCT	ADHF, PAWP ≥15 mmHg, and cardiac index <2.5 L/min/m^2^	TEZ (50^a^ and 100 mg/h)		90/191	Placebo	94
CIN	Erdmann *et al*.^15^	DB RCT	ADHF, PAWP ≥18 mmHg, and LVEF <40%	CIN (100 μg/h)		97/97	Placebo	51
SER	Ponikowski *et al*.^16^	DB RCT	ADHF, PAWP ≥18 mmHg, GFR ≥30 mL/min/1.73 m^2^, and SBP ≥115 mmHg	SER (30 μg/kg/day)		34/34	Placebo	37

ADHF, acute decompensated heart failure; CHF, congestive heart failure; CIN, cinaciguat; DB RCT, double‐blind randomized controlled trial; GFR, glomerular filtration rate; HF, heart failure; LEV, levosimendan; LEVp, levosimendan pilot study; LIDO, Levosimendan Infusion vs. Dobutamine; LVEF, left ventricular ejection fraction; NES, nesiritide; NESp, nesiritide pilot study; NYHA, New York Heart Association; PAWP, pulmonary arterial wedge pressure; SBP, systolic blood pressure; SER, serelaxin; SIRIUS II, Safety and efficacy of an Intravenous placebo‐controlled Randomized Infusion of Ularitide in a prospective double‐blind Study; TEZ, tezosentan; TEZhd, tezosentan high dose; TEZld, tezosentan low dose; TEZp, tezosentan pilot study; total, total number of eligible patients who received study treatment; ULA, ularitide; ULAp, ularitide pilot study; VERITAS, Value of Endothelin Receptor Inhibition With Tezosentan in Acute Heart Failure Studies; VMAC, Vasodilatation in the Management of Acute CHF.

a Dose selected for meta‐analysis.

b Other study arm was nitroglycerin (*n* = 60) for 3 h (not used).

c Dose at the investigator's discretion.

d Other study arms were dobutamine (6 μg/kg/min) and ethanol vehicle open‐label exploratory comparators (not used).

Studies were categorized into seven ‘main’ studies and four ‘pilot’ studies (Supporting Information, *Table* *S3*). Main studies were placebo controlled (≤3 h, *n* = 6; >3 h, *n* = 5) or active controlled (≤3 h, *n* = 1; >3 h, *n* = 2); all pilot studies were placebo controlled. In total, 622 patients were included for the 3 h analysis and 644 for the 6 and 24 h analyses. The comparator groups consisted of 520 and 543 patients, respectively.

Baseline characteristics are summarized in *Table* 2 for the controlled main studies.

**Table 2:** Baseline characteristics in the controlled main studies (means ± standard deviations or frequencies, respectively, in the pooled IMP and comparator groups)

Parameter	ULA	NES	LEV	TEZld	TEZhd	CIN	SER
*n*	106^a^	246	203	84^b^	285^c^	148^d^	71^e^
Age (years)	60 ± 12	–f	59 ± 11	–f	61 ± 13	62 ± 11	69 ± 12
Male (%)	76	–f	87	–f	79	85	75
HR (/min)	76 ± 12	–	82 ± 16	–f	–	80 ± 14	76 ± 17
PAWP (mmHg)	25 ± 6	28 ± 7	25 ± 8	26 ± 6	25 ± 7	25 ± 5	26 ± 6
Cardiac index (L/min/m^2^)	1.90 ± 0.35	2.18 ± 0.73	1.93 ± 0.40	2.08 ± 0.49	1.93 ± 0.36	2.16 ± 0.60	2.30 ± 0.65
RAP (mmHg)	10 ± 5	−15 ± 7	−10 ± 7	15 ± 7	–	12 ± 5	13 ± 6
SBP (mmHg)	126 ± 19	121 ± 22	114 ± 18	–f	–	123 ± 17	131 ± 16
SVR (dyn·s/cm^5^)	1863 ± 512	1443 ± 611	1959 ± 565	1778 ± 678	–	1605 ± 524	1623 ± 538

–, not shown; CIN, cinaciguat; HR, hazard ratio; IMP, investigational medical product; LEV, levosimendan; NES, nesiritide; PAWP, pulmonary arterial wedge pressure; RAP, right atrial pressure; SBP, systolic blood pressure; SER, serelaxin; SVR, systemic vascular resistance; TEZhd, tezosentan high dose; TEZld, tezosentan low dose; ULA, ularitide.

a HR, PAWP, cardiac index, RAP, SBP (*n* = 104), and SVR (*n* = 100).

b RAP (*n* = 83).

c PAWP (*n* = 256).

d PAWP, cardiac index, RAP, and SVR (*n* = 139).

e HR, PAWP, cardiac index, RAP, and SVR (*n* = 63).

f Values not available for the subgroup of catheterized patients.

### Risk of bias within studies

Sequence generation, allocation concealment, and blinding of study arms were regarded as adequate in all studies except VERITAS.^14^ In VERITAS, the low‐dose tezosentan arm was a small subgroup of a larger trial, with no information provided on stratified randomization.^14^ Outcome data were incompletely reported (missing data on patient disposition) in VERITAS,^14^ Nieminen *et al*.,^24^ and Erdmann *et al*.^15^ In VMAC,^17^ complete presentation of the initial study phase (3 h) is available (nesiritide vs. nitroglycerin vs. placebo), but the subsequent study phase (>3 h; nesiritide vs. nitroglycerin) was selectively reported. In the LIDO study,^13^ PAWP was the only endpoint suitable for the meta‐analysis.

### Heterogeneity assessment

Studies with ularitide yielded *I* ^2^ of 0% in all analyses. While this is obviously true if only one study is considered, because the distinction between the fixed‐effects and random‐effects models has no meaning, no heterogeneity occurred when both ularitide studies were included, either. The synthesis of other studies showed several cases of heterogeneity indicating differences between the effect sizes of the other study treatments. Significant and quantitatively high heterogeneity was observed for cardiac index and SVR after 6 h. The primary analysis in all controlled main studies showed a moderate (~50%) but insignificant heterogeneity. No heterogeneity was detected for PAWP in placebo‐controlled main studies at 6 h (Supporting Information, *Table* *S4*).

### Course of haemodynamic parameters in the controlled main studies

**Figure 2:**
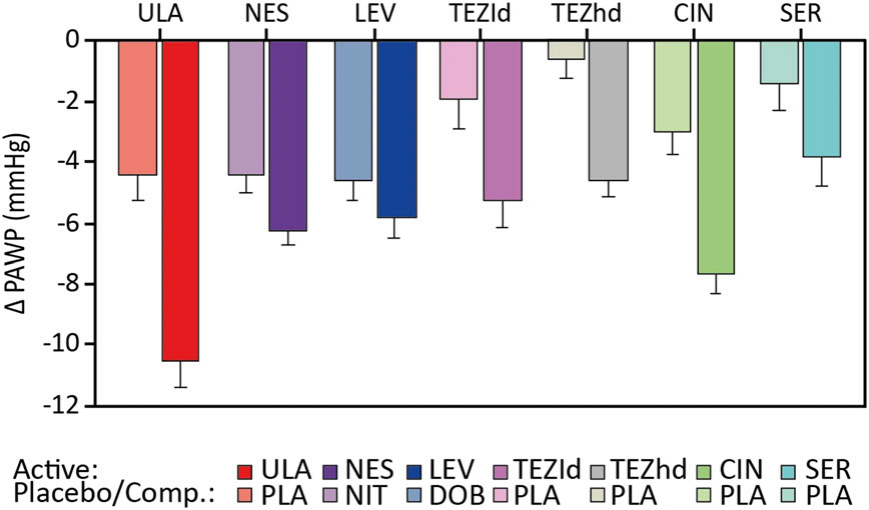
Mean changes in pulmonary arterial wedge pressure (PAWP) at 6 h with ularitide vs. placebo and other agents vs. comparator (Comp.; other active treatment or placebo). Bars are the mean with standard error. CIN, cinaciguat; DOB, dobutamine; LEV, levosimendan; NES, nesiritide; NIT, nitroglycerin; PLA, placebo; SER, serelaxin; TEZhd, tezosentan high dose; TEZld, tezosentan low dose; ULA, ularitide.

Change in PAWP at 6 h with ularitide vs. placebo was numerically greater than with other agents vs. comparators (*Figure* *2*). Similar but less marked differences between agents were seen at 3 and 24 h (Supporting Information, *Figure* *S1* *A*). Changes in cardiac index at 3, 6, and 24 h varied widely between compounds and were greatest with cinaciguat (Supporting Information, *Figure* *S1* *B*).

**Figure 3:**
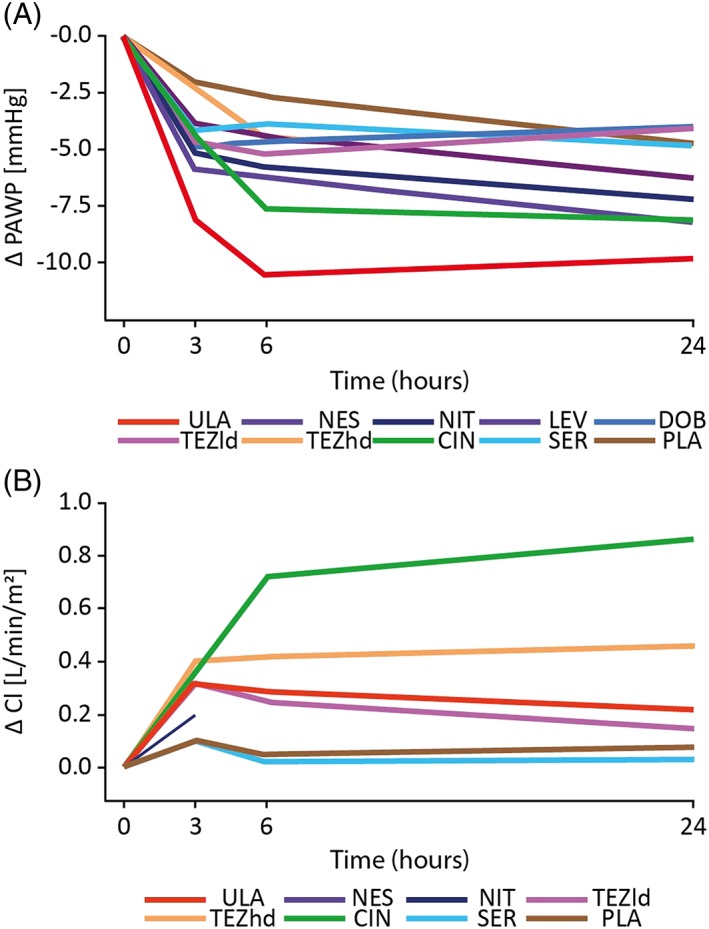
Mean 24 h changes in pulmonary arterial wedge pressure (PAWP) and cardiac index (CI). Time course of mean changes from baseline to 24 h in (A) PAWP and (B) CI. CIN, cinaciguat; DOB, dobutamine; LEV, levosimendan; NES, nesiritide; NIT, nitroglycerin; PLA, placebo; SER, serelaxin; TEZhd, tezosentan high dose; TEZld, tezosentan low dose; ULA, ularitide.

In controlled main studies, PAWP improved after 3 and 6 h of active treatment and generally remained stable or declined slightly after 24 h (*Figure* *3* *A*). The placebo curve, calculated by meta‐analytical synthesis of all placebo curves from placebo‐controlled clinical trials, was located in a low‐effect region of changes from baseline in PAWP. Thus, significant differences between study drug and comparator were generally observed after 6 h (except for levosimendan vs. dobutamine and serelaxin vs. placebo; *Figure* *3* *A*). Cardiac index increased (*Figure* *3* *B*), and RAP, SBP, DBP, and SVR decreased after 3–6 h of treatment vs. placebo or active comparator.

### Meta‐analysis of pulmonary arterial wedge pressure at 6 h

**Figure 4:**
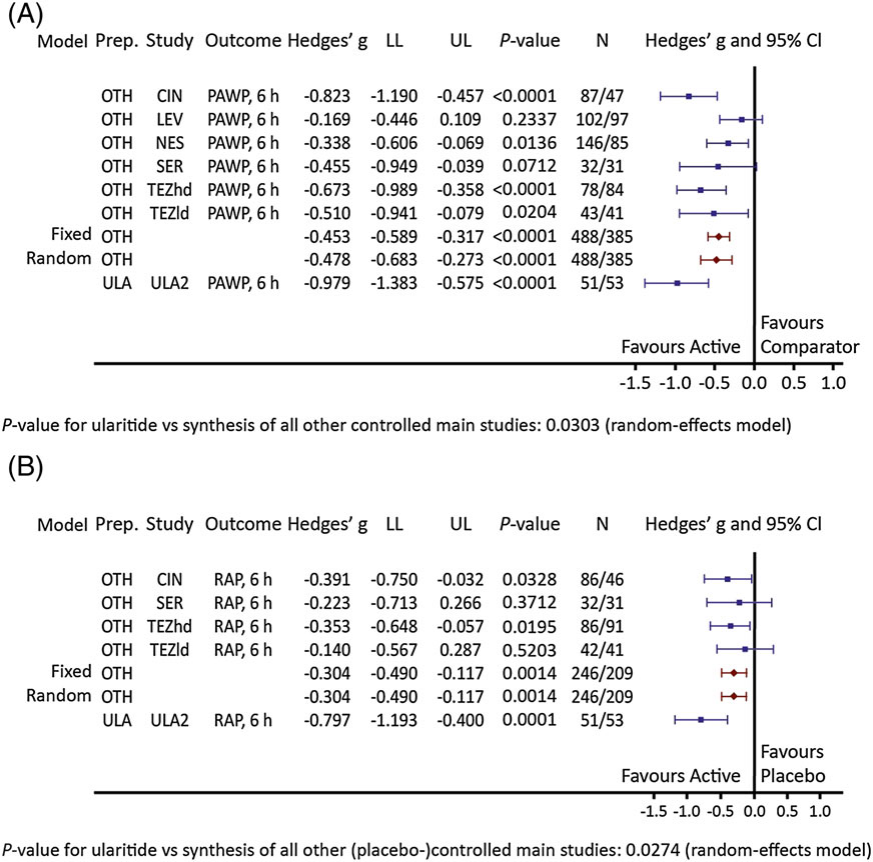
Pulmonary arterial wedge pressure (PAWP) and right atrial pressure (RAP) effect sizes for ularitide vs. other controlled main studies. Hedges' *g* scores [95% confidence intervals (CIs)] of changes from baseline to 6 h in (A) PAWP and in (B) RAP in the ularitide study Safety and efficacy of an Intravenous placebo‐controlled Randomized Infusion of Ularitide in a prospective double‐blind Study (ULA2)^1^ and the synthesis of all other controlled main studies (OTH). CIN, cinaciguat; LEV, levosimendan; LL, lower limit; NES, nesiritide; SER, serelaxin; TEZhd, tezosentan high dose; TEZld, tezosentan low dose; ULA, ularitide; UP, upper limit.

Meta‐analysis of changes from baseline in PAWP after 6 h of treatment with ularitide vs. placebo in the SIRIUS II study^1^ produced a Hedges' *g* effect size of −0.979 (*P* < 0.0001). With the other compounds vs. comparators in controlled main studies, the Hedges' *g* effect size was −0.478 (*P* < 0.0001) (*Figure* *4* *A*). The treatment difference between ularitide compared with the synthesis of all other controlled main studies [−0.501; 95% confidence interval (CI): −0.954, −0.048] was statistically significant (*P* = 0.0303).

### Meta‐analyses of pulmonary arterial wedge pressure at 3 and 24 h

Effect sizes were higher with ularitide at 3 h (Hedges' *g*, −0.816; *P* < 0.0001) and 24 h (−0.610; *P* = 0.0022) vs. all other treatments (−0.421; *P* = 0.0004 and − 0.342; *P* < 0.0001, respectively) in controlled main studies. Treatment differences at 3 h (−0.395; 95% CI: −0.857, 0.068; *P* = 0.0944) and 24 h (−0.268; 95% CI: −0.691, 0.154; *P* = 0.2132) were not statistically significant (Supporting Information, *Figure S2*).

Sensitivity meta‐analyses of primary and secondary haemodynamic endpoints are detailed in Supporting Information, *Appendix S1*.

### Meta‐analyses of additional haemodynamic endpoints in placebo‐controlled main studies

Additional haemodynamic endpoints were analysed only in placebo‐controlled main studies, as they were not reported in the two active‐controlled studies.

After 6 h, a significant increase in cardiac index and significant decreases in RAP, SBP, DBP, and SVR were observed, with somewhat larger effect sizes for ularitide vs. placebo compared with the pooled effect sizes for other study treatments vs. placebo. A significant difference was seen only for RAP (Hedges' *g*, −0.797 for ularitide and −0.304 for other treatments; *P* = 0.0274 for ularitide vs. synthesis of all other (placebo‐)controlled main studies; *Figure* *4* *B*). Further details are shown in Supporting Information, *Table* *S5*.

### Meta‐analyses of other endpoints

The BNP/NT‐proBNP cluster analysis showed no statistically significant changes from baseline after 6 h of treatment, although there was a parallel significant decrease after 24 h with both ularitide and other treatments (Supporting Information, *Table* *S6*). Serum creatinine showed slight changes from baseline after 24 h (Supporting Information, *Table* *S6*); in the analysis of other treatments, this was the result of contrary results with levosimendan (Hedges' *g*, −0.512; *P* = 0.0006) and tezosentan (Hedges' *g*, 0.452; *P* = 0.0024).

There was no evidence of increased mortality in placebo‐controlled main studies. The risk ratio for ularitide vs. placebo (0.286; 95% CI: 0.062, 1.312; *P* = 0.1073) was lower than for other placebo‐controlled main studies (0.994; 95% CI: 0.428, 2.307; *P* = 0.9890), although the difference was not statistically significant (*P* = 0.1606; Supporting Information, *Figure S3*).

### Meta‐analyses of safety results

In placebo‐controlled main studies, no significant treatment differences between ularitide and other treatments were detected in terms of infusion discontinuation, infusion discontinuation due to adverse events, or incidences of adverse events or serious adverse events during infusion (Supporting Information, *Table* *S7*).

## Discussion

In this meta‐analysis, PAWP—the primary endpoint in the trials and a proxy for left ventricular end‐diastolic pressure—was reduced significantly by ularitide vs. placebo. There were numerical improvements with ularitide vs. other pooled study treatments, with a significant difference at 6 h in controlled main studies. The magnitude of improvement with ularitide in SIRIUS II^1^ was greater than in the other individual main studies (*Figure* *2*).^1^, ^13^, ^14^, ^15^, ^16^, ^17^, ^25^ Right atrial pressure (a proxy for right ventricular end‐diastolic pressure) also showed favourable effects for ularitide vs. all other treatments pooled. Effects on other haemodynamic parameters showed no significant differences between ularitide and other study treatments at any time point. Although the comparator substances in this meta‐analysis vary in their mechanisms of action, all produce primarily vasodilatation without positive inotropic effects. The authors therefore believe that comparisons between them are possible.

The results of the present study are consistent with previous meta‐analyses of vasoactive drugs. A meta‐analysis comparing vasodilators and inotropes showed that the two classes reduce left‐sided and right‐sided filling pressures to a similar degree in patients with acute heart failure and reduced left ventricular ejection fraction.^26^

While PAWP can be considered as a proxy for left ventricular end‐diastolic pressure (LVEDP), it must be emphasized that patients with high‐grade mitral regurgitation or clinically relevant mitral stenosis were excluded from the trials so that it can be assumed that PAWP correlates with LVEDP. There are no data available on the percentage of patients with atrial fibrillation in the included studies so that a corresponding analysis could not be performed.

Safety was not a main endpoint of this analysis, although ularitide seems to have similar tolerability to the comparators in terms of discontinuations and infusion‐related adverse events, with no signal of increased mortality with any treatment. Safety data in the current study should be viewed with caution because of the limited size and duration of the included studies and the large CIs of the estimates. In a meta‐analysis of drugs and medical treatment algorithms, the authors concluded that ularitide is a promising novel therapy for ADHF.^27^ If confirmed, the combination of haemodynamic efficacy and good tolerability with ularitide would suggest a favourable risk–benefit profile.

Mortality in Phase 2 studies was generally lower in patients receiving active treatment vs. controls, leading to the development of Phase 3 trials, although no definitive improvement of mortality could be shown. This was also the case in two Phase 3 trials presented or published after the present analysis was conducted. In the Trial of Ularitide Efficacy and Safety in Acute Heart Failure study, 2157 patients with AHF received standard therapy plus either a continuous intravenous infusion of ularitide 15 ng/kg/min or matching placebo for 48 h.^11^ Compared with placebo, ularitide reduced systolic blood pressure and NT‐proBNP (considered a marker of left ventricular pressure/volume overload) and favourably influenced markers of congestion such as haemoglobin and transaminases. There were, however, no significant differences between treatment arms in death from cardiovascular causes during median follow‐up of 15 months nor in a hierarchical composite endpoint evaluating disease course during the 48 h treatment period. The absence of significant prognostic benefit appears to be the result of enrolment of ineligible patients, as a *post hoc* analysis excluding patients identified as ineligible for the trial suggested a reduction in the hierarchical endpoint with ularitide.^11^ In the serelaxin in addition to standard therapy in acute heart failure study, ~6600 patients hospitalized for AHF were randomized to standard care plus either 48 h intravenous infusion of serelaxin (30 μg/kg/day) or placebo.^28^

There was no significant difference between serelaxin and placebo with regard to either co‐primary endpoint: 180 day cardiovascular death and worsening heart failure through Day 5. Nesiritide was reported to improve readmission rates and survival compared with dobutamine in a meta‐analysis^29^ but did not reduce mortality in the placebo‐controlled Phase 3 ASCEND‐HF study^30^ or a later meta‐analysis.^31^ A Phase 3 mortality trial involving cinaciguat was terminated prematurely because of a high incidence of hypotension.^15^ Overall, therefore, there is no clear evidence for the optimal treatment to improve prognosis in ADHF. None of the vasoactive agents examined in the present analysis have a level of recommendation 1A for intravenous administration, so the search for new substances with different mechanisms of action is ongoing.

There are several possible reasons why the favourable haemodynamic effects obtained in proof‐of‐concept Phase 2 studies, as well as improvements in other surrogate markers such as NT‐proBNP, haemoglobin, and transaminases in some studies, did not result in a significant decrease of mortality after approximately 180 days during the Phase 3 trials. These include short duration of infusion time (24–48 h), fixed treatment dose regimen, protocol violations and the failure to use guideline‐directed therapy in the majority of Phase 2 trials. The hypothesis underlying the use of vasoactive substances in ADHF—that rapid reversal of ventricular wall stress preserves myocardial viability—may itself be plausible, but cannot be translated into a mortality benefit.^11^ Another possibility is that the reduction of micro‐myocardial injury by unloading the ventricle is insufficient to influence long‐term outcomes.^11^

There are numerous challenges in conducting clinical trials in acute heart failure.^32^ Many clinicians have rejected the expressions ‘systolic heart failure’ and ‘diastolic heart failure’ in favour of ‘heart failure with reduced ejection fraction’ and ‘heart failure with preserved ejection fraction’ because they describe the results of cardiac function testing, and abnormal systole and diastole can occur in the same patient. Pathophysiology of this condition, which can include myocardial ischaemia, arrhythmia, valve dysfunction, or volume overload, is poorly understood, and for many years it was not recognized as a distinct entity. Lack of objective diagnostic criteria means that populations are heterogeneous, making it difficult to develop clear inclusion criteria. This is complicated by differing regulatory requirements for trials in acute heart failure between countries. There is no agreement among physicians and decision makers on therapy goals and trial endpoints in ADHF, and no clear links between haemodynamic endpoints and clinical outcomes. ADHF of differing aetiology may not respond in the same way to a given intervention. Haemodynamic parameters are not a reliable guide to dosing of novel therapies, although some can act as surrogates for symptoms (e.g. PAWP and dyspnoea). Comparisons across studies are complicated by different timing and duration of interventions, as well as differing definitions of ‘standard therapy’. It is unclear whether novel therapies should be evaluated as monotherapy or as add‐ons, particularly given the relatively low cost of conventional therapies.

Administration of intravenous diuretics (furosemide 40–80 mg) before initiation of study therapy was mandatory in the trials included in this analysis. No intravenous administration of diuretics was allowed during the study unless the patients showed a clinically relevant haemodynamic deterioration (worsening heart failure). There are no randomized haemodynamic placebo‐controlled trials with diuretics, and therefore, the effects of concomitant diuretics on the endpoints studied here cannot be ascertained.

### Limitations of the meta‐analysis

The key limitation of the present meta‐analysis is the small number of studies available and their small size and short duration. Much of the data were extracted from published graphs by digitization (Supporting Information, *Appendix S1*). Based on the included studies, there were insufficient data to analyse several additional outcomes of interest, including pulmonary vascular resistance, the percentage of patients with an increased pre‐capillary pulmonary artery pressure, pressure gradients between PAWP and the pulmonary vasculature, and oxygen saturation in the systemic and pulmonary circulations. Pulmonary vascular resistance was not available for the ularitide studies, and therefore, we cannot compare its effects on this parameter with other vasoactive substances. Similarly, we cannot speculate on the effects of ularitide on the pulmonary vs. the systemic circulation. According to the original publications, other vasoactive substances reduce pulmonary vascular resistance and SVR, with no preferential effect on the pulmonary circulation. The present publications had no data on oxygen saturation within the systemic and pulmonary circulations. Consequently, the role of hypoxic pulmonary vasoconstriction in the results observed is not known. Furthermore, only patients with heart failure and reduced ejection fraction were enrolled in the included studies so that no extrapolation of data on heart failure with preserved ejection fraction is possible. Finally, no correction for multiple comparisons was carried out as part of the statistical analysis.

There is currently no international consensus on how to measure PAWP, which can be measured in end‐expiratory apnoea or during quiet respiration at a specified point before the inspiratory dip of the pressure curve.^33^, ^34^ This is, however, extremely difficult in patients with Cheyne–Stokes respiration and in atrial fibrillation. The most common practice, especially in the intensive care units and cardiac catheterization laboratories, is to use a computer‐generated pressure measurement during quiet breathing over a period of 5 s. These computer‐generated wedge pressure measurements are consistently and significantly lower than the results obtained during expiration. An accurate haemodynamic manual exists for the trials with ularitide, levosimendan, cinaciguat, and serelaxin. In all these trials, PAWP was measured at the end of expiration during slight apnoea while the patient's mouth was open to avoid Valsalva pressing. In other trials, such as those with tezosentan and nesiritide, PAWP was measured during expiration.

Furthermore, there is also no consensus among experts on how to perform levelling and determination of the zero point. According to several publications, the zero point or zero reference level is generally recommended to be set at the level of the right atrium or tricuspid valve.^35^, ^36^ In practice, the most frequently used zero level in the supine patient is at mid‐thoracic level or at one‐third of the thoracic diameter below the anterior thorax surface. According to the computed tomography study performed by Kovacz, one‐third of the thoracic diameter mostly represents the right atrium, while the left atrium is represented best by the mid‐thoracic level.^37^ In the studies performed with ularitide, levosimendan, serelaxin, and cinaciguat, the zero level was set at one‐third of the thoracic diameter below the anterior thorax surface, as per the haemodynamic manuals. In the trials with tezosentan and nesiritide, the zero level was set at the mid‐thoracic level. Baseline levels of PAWP in all these trials were similar, regardless of how PAWP was measured and how levelling was performed so that it was possible to analyse differences between the drugs.

In conclusion, ularitide demonstrated high effect sizes with respect to PAWP and RAP after 6 h of treatment. The haemodynamic improvements seen with ularitide, combined with its beneficial effects on renal function, dyspnoea, myocardial structure, and endothelin levels,^8^ suggest that ularitide may be a promising drug for recompensation of patients with ADHF.

## Conflict of interest

V.M. and S.B.F. have received consultation fees and honoraria from Bayer Healthcare AG, Novartis Pharma, and Cardiorentis Ltd.

## Funding

This study, including design and conduct; collection, management, analysis, and interpretation of the data; preparation, review, and approval of the manuscript; and decision to submit the manuscript for publication, was funded and supported entirely by PHARIS Biotec GmbH.

## Supporting information


**Table S1.** Search terms for: a) Cochrane Central Register of Controlled Trials (DIMDI); b) MEDLINE (DIMDI); c) MEDLINE (PubMed); d) ClinicalTrials.gov.

**Table S2.** Parameters used for meta‐analysis per study and per target time of control after baseline (3, 6, and 24 hours). a) Haemodynamic parameters; b) Laboratory parameters; c) Binary parameters.

**Table S3.** Number of patients in the selected dose groups of the investigational medical product (IMP) and the comparator product (Comp.) stratified into main and pilot studies.

**Table S4.** Heterogeneity in the OTHER studies: a) Controlled main studies; b) Placebo‐controlled main studies; c) All controlled studies.

**Table S5.** Hedges' *g* scores (95% CIs) of haemodynamic parameters for ularitide vs. placebo and the synthesis of all other study treatments vs. placebo (random‐effects model; placebo‐controlled main studies).

**Table S6**. Hedges' *g* scores (95% CIs) of other endpoints for the synthesis of ularitide vs. placebo and the synthesis of all other study treatments vs. comparator (random‐effects model; placebo‐controlled main studies).

**Table S7**. Risk ratios (95% CIs) of safety results for ularitide vs. placebo and the synthesis of all other treatments vs. placebo (random‐effects model; placebo‐controlled main studies).

**Figure S1**. Mean changes in: a) PAWP; b) cardiac index at 3, 6, and 24 hours with active compounds. Bars are the mean with standard error. CIN, cinaciguat; LEV, levosimendan; PAWP, pulmonary arterial wedge pressure; NES, nesiritide; NIT, nitroglycerin; SER, serelaxin; TEZhd, tezosentan high dose; TEZld, tezosentan low dose; ULA, ularitide.
**Figure S2**. Hedges' *g* scores (95% CIs) of changes from baseline to: a) 3 hours; b) 24 hours in pulmonary arterial wedge pressure in the ularitide study SIRIUS II (ULA)^1^ and the synthesis of all other controlled main studies (OTH). CI, confidence interval; CIN, cinaciguat; LEV, levosimendan; LL, lower limit; NES, nesiritide; NIT, nitroglycerin; SER, serelaxin; TEZhd, tezosentan high dose; TEZld, tezosentan low dose; UL, upper limit; ULA, ularitide.
**Figure S3**. Risk ratios (RRs) (95% CIs) of 30‐day mortality rates in the ularitide study SIRIUS II (ULA)^1^ and the synthesis of the placebo‐controlled main studies. CI, confidence interval; CIN, cinaciguat; LL, lower limit; SER, serelaxin; TEZhd, tezosentan high dose; UL, upper limit; ULA, ularitide.
**Figure S4**. Sensitivity analysis. Hedges' *g* scores (95% CIs) of changes from baseline to: a) 3 hours; b) 6 hours; c) 24 hours in pulmonary arterial wedge pressure in the ularitide study SIRIUS II (ULA)^1^ and the synthesis of all other placebo‐controlled main studies (OTH). CI, confidence interval; CIN, cinaciguat; LEV, levosimendan; LL, lower limit; NES, nesiritide; PAWP, pulmonary arterial wedge pressure; SER, serelaxin; TEZhd, tezosentan high dose; TEZld, tezosentan low dose; UL, upper limit; ULA, ularitide.
**Figure S5**. Sensitivity analysis: Hedges' *g* scores (95% CIs) of changes from baseline to: a) 3 hours; b) 6 hours; c) 24 hours in pulmonary arterial wedge pressure in the ularitide studies SIRIUS I^8^ and II (ULAp, ULA)^1^ and the synthesis of all other controlled pilot and main studies (OTH). CI, confidence interval; CIN, cinaciguat; LEV, levosimendan; LL, lower limit; NES, nesiritide; PAWP, pulmonary arterial wedge pressure; SER, serelaxin; TEZhd, tezosentan high dose; TEZld, tezosentan low dose; UL, upper limit; ULA, ularitide.

Supporting info item

Supporting info item

Supporting info item

Supporting info item

Supporting info item
