# Supplementary material for: Randomized double‐blind clinical studies of ularitide and other vasoactive substances in acute decompensated heart failure: a systematic review and meta‐analysis
Source: ESC Heart Fail. 2018 Sep 24;5(6):1023–34. doi: 10.1002/ehf2.12349 (PMC6300812; doi:10.1002/ehf2.12349)
Supplement: Supplementary file 1 — Table S1. Search terms for: a) Cochrane Central Register of Controlled Trials (DIMDI); b) MEDLINE (DIMDI); c) MEDLINE (PubMed); d) ClinicalTrials.gov. [file EHF2-5-1023-s001.docx]

**Table S1. Search Terms for: a) Cochrane Central Register of Controlled Trials (DIMDI); b) MEDLINE (DIMDI); c) MEDLINE (PubMed); d) ClinicalTrials.gov**

**a)**

| **#** | **Search Term** | **Hits** |
| --- | --- | --- |
| 1 | CCTR93 | 856 305 |
| 2 | ((ACUTE OR DECOMPENSATED OR CONGESTIVE) AND (HEART FAILURE)) OR (ACUTE HEART DECOMPENSATION) OR (ACUTE DECOMPENSATION AND HEART) OR (LOW-OUTPUT AND HEART AND FAILURE) OR ADHF OR AHF OR HHF OR AHFS OR DHF OR CHF | 6001 |
| 3 | (CLINICAL AND TRIALS,RANDOMISED) OR (CONTROLLED AND CLINICAL AND TRIALS, RANDOMISED) OR RCT OR (RANDOMISED AND CONTROLLED AND TRIAL) OR (CLINICAL AND STUDY, RANDOMISED) OR (CONTROLLED AND CLINICAL AND STUDY, RANDOMISED) OR (RANDOMISED AND CONTROLLED AND STUDY) | 612 854 |
| 4 | ULARITIDE OR URODILATIN OR NESIRITIDE OR NATRECOR OR LEVOSIMENDAN OR SIMADAX OR DEXTROSIMENDAN OR TEZOSENTAN OR VELETRI OR CINACIGUAT OR BAY582667 OR RELAXIN OR SERELAXIN | 427 |
| 5 | (PULMONARY AND CAPILLARY AND WEDGE AND PRESSURE) OR **PAWP** OR (SYSTEMIC AND VASCULAR AND RESISTANCE) OR SVR OR (CARDIAC AND INDEX) OR CI OR (RIGHT AND ATRIAL AND PRESSURE) OR RAP OR (MEAN AND ARTERIAL AND PRESSURE) OR MAP OR SBP OR DBP OR (LOW AND CARDIAC AND OUTPUT) OR (HEMODYNAMIC AND EFFECT) OR (HAEMODYNAMIC AND EFFECT) OR (STROKE AND VOLUME AND INDEX) OR (EJECTION AND FRACTION) OR (VENTRICULAR AND EJECTION AND FRACTION) OR (STROKE AND VOLUME) | 65 005 |
| 6 | 2 AND 3 AND 4 AND 5 | 117 |
| 7 | Check duplicates: unique in s = 6 | 114 |
|  | Sort parameter: AU/A; PY/A |  |

**b)**

| **#** | **Search Term** | **Hits** |
| --- | --- | --- |
| 1 | ME60 | 25 035 328 |
| 2 | ((ACUTE OR DECOMPENSATED OR CONGESTIVE) AND (HEART FAILURE)) OR (ACUTE HEART DECOMPENSATION) OR (ACUTE DECOMPENSATION AND HEART) OR (LOW-OUTPUT AND HEART AND FAILURE) OR ADHF OR AHF OR HHF OR AHFS OR DHF OR CHF | 64 309 |
| 3 | (CLINICAL AND TRIALS, RANDOMISED) OR (CONTROLLED AND CLINICAL AND TRIALS, RANDOMISED) OR RCT OR (RANDOMISED AND CONTROLLED AND TRIAL) OR (CLINICAL AND STUDY, RANDOMISED) OR (CONTROLLED AND CLINICAL AND STUDY, RANDOMISED) OR (RANDOMISED AND CONTROLLED AND STUDY) | 494 873 |
| 4 | ULARITIDE OR URODILATIN OR NESIRITIDE OR NATRECOR OR LEVOSIMENDAN OR SIMADAX OR DEXTROSIMENDAN OR TEZOSENTAN OR VELETRI OR CINACIGUAT OR BAY582667 OR RELAXIN OR SERELAXIN | 4652 |
| 5 | (PULMONARY AND CAPILLARY AND WEDGE AND PRESSURE) OR **PAWP** OR (SYSTEMIC AND VASCULAR AND RESISTANCE) OR SVR OR (CARDIAC AND INDEX) OR CI OR (RIGHT AND ATRIAL AND PRESSURE) OR RAP OR (MEAN AND ARTERIAL AND PRESSURE) OR MAP OR SBP OR DBP OR (LOW AND CARDIAC AND OUTPUT) OR (HEMODYNAMIC AND EFFECT) OR (HAEMODYNAMIC AND EFFECT) OR (STROKE AND VOLUME AND INDEX) OR (EJECTION AND FRACTION) OR (VENTRICULAR AND EJECTION AND FRACTION) OR (STROKE AND VOLUME) | 684 755 |
| 6 | 2 AND 3 AND 4 AND 5 | 130 |
| 7 | Check duplicates: unique in s = 6 | 130 |
|  | Sort Parameter: AU/A; PY/A |  |

**c)**

| **#** | **Search Term** | **Hits** |
| --- | --- | --- |
| q100 | “(((acute or congestive) and heart and failure) OR  (acute Heart Decompensation or acute Decompensation,Heart) OR low-output+heart+failure [All Fields] OR ADHF[All Fields] OR  AHF[All Fields] OR HHF[All Fields] OR AHFS[All Fields] OR  DHF[All Fields] OR CHF[All Fields])" | 70 703 |
| q101 | "((clinical+trials,randomised[TW] OR controlled+clinical+trials,randomised[TW] OR RCT[TW] OR clinical+study,randomised[TW] OR controlled+clinical+study,randomised[TW] OR randomised+controlled+trial[TW] OR randomised+controlled+study[TW]))" | 410 629 |
| q200 | "(ularitide[TW] OR urodilatin[TW] OR nesiritide[TW] OR natrecor[TW] OR  levosimendan[TW] OR (Simadax or dextrosimendan or OR 1259 or OR-1259) OR tezosentan[TW] OR (RO 61-0612 or RO 610612 or Veletri) OR cinaciguat[TW] or BAY582667[TW] OR relaxin[TW] or serelaxin[TW])" | 9703 |
| q300 | "(Pulmonary+Capillary+Wedge+Pressure[MeSH Terms] OR **PAWP**[MeSH Terms] OR systemic+vascular+resistance[MeSH Terms] OR SVR[MeSH Terms] OR  Cardiac+index [MeSH Terms] OR CI[MeSH Terms] OR right+atrial+pressure [MeSH Terms] OR RAP [MeSH Terms] OR Mean+arterial+pressure [MeSH Terms] OR MAP[MeSH Terms] OR SBP[MeSH Terms] OR DBP[MeSH Terms] OR low+cardiac+output [MeSH Terms] OR hemodynamic+effect [MeSH Terms] OR  (stroke+volume+index [MeSH Terms] OR ejection+fraction [All Fields] OR  Ventricular+Ejection+Fraction[MeSH Terms] or Stroke+Volume[MeSH Terms]))" | 106 182 |
| Total | Unique(q100 AND q101 AND q200 AND q300) | 50 |

**d)**

| **#** | **Search Term** | **Hits** |
| --- | --- | --- |
| q100 | "('Acute Heart Failure (AHF)' OR 'Acute Heart Failure' OR 'Heart Failure, Acute' OR 'Heart Failure, Congestive' OR 'Congestive Heart Failure' OR 'Acute Decompensated Heart Failure' OR 'low-output heart failure')" | 4069 |
| q101 | "('clinical trials,randomised' OR 'controlled clinical trials,randomised' OR 'clinical study,randomised' OR 'controlled clinical study,randomised' OR 'randomised controlled trial' OR 'randomised controlled study')" | 103 014 |
| q200 | "('Ularitide' OR 'Urodilatin' OR 'Nesiritide' OR 'Natrecor' OR 'Levosimendan' OR 'Tezosentan' OR 'Cinaciguat' OR 'Relaxin' OR 'Serelaxin')" | 620 |
| q300 | "('Pulmonary Capillary Wedge Pressure' OR 'PCWP' OR 'systemic vascular resistance' OR 'SVR' OR 'Cardiac index' OR 'CI' OR 'right atrial pressure' OR 'RAP' OR 'Mean arterial pressure' OR 'MAP' OR 'SBP' OR 'DBP' OR 'low cardiac output' OR 'hemodynamic effect' OR 'stroke volume index')" | 7889 |
| Total | Unique(paste(q100, q101, q200, q300, sep=' AND ')) | 75 |
|  | No further filters set such as recruiting, not yet recruiting, active etc. |  |

ADHF, acute decompensated heart failure; AHF, acute heart failure; AHFS, acute heart failure syndrome; CHF, congestive heart failure; CI, cardiac index; DBP, diastolic blood pressure; DHF, decompensated heart failure; DIMDI, Deutsches Institut für Medizinische Dokumentation und Information; HHF, hospitalised heart failure; MAP, mean arterial pressure; MeSH, Medical Subject Heading; **PAWP, pulmonary arterial wedge pressure;** PCWP, pulmonary capillary wedge pressure; RAR, right atrial pressure; RCT, randomized controlled trial; SBP, systolic blood pressure; SVR, systemic vascular resistance.
