# Supplementary material for: Randomized double‐blind clinical studies of ularitide and other vasoactive substances in acute decompensated heart failure: a systematic review and meta‐analysis
Source: ESC Heart Fail. 2018 Sep 24;5(6):1023–34. doi: 10.1002/ehf2.12349 (PMC6300812; doi:10.1002/ehf2.12349)
Supplement: Supplementary file 2 — Table S2. Parameters used for meta‐analysis per study and per target time of control after baseline (3, 6, and 24 hours). a) Haemodynamic parameters; b) Laboratory parameters; c) Binary parameters. [file EHF2-5-1023-s002.docx]

**Table S2.** **Parameters used for meta-analysis per study and per target time of control after baseline (3, 6, and 24 hours). a) Haemodynamic parameters; b) Laboratory parameters; c) Binary parameters**

**a)**

| **Parameter** | **Time (hours)** | **ULAp** | **ULA** | **NESp** | **NES ≤3 h** | **NES >3 h** | **LEVp** | **LEV** | **TEZp** | **TEZld** | **TEZhd** | **CIN** | **SER** |
| --- | --- | --- | --- | --- | --- | --- | --- | --- | --- | --- | --- | --- | --- |
| **PAWP** | 3 | – | X | X | X |  | X | X | X | X | – | X | X |
|  | 6 | X | X | X |  | X | X | X | X | X | X | X | X |
|  | 24 | X | X | X |  | X | X | X | X | X | – | X | X |
| CI | 3 | – | X | X | X |  | – | – | X | X | X | X | X |
|  | 6 | X | X | X |  | – | – | – | X | X | X | X | X |
|  | 24 | X | X | X |  | – | – | – | X | X | X | X | X |
| RAP | 3 | – | X | X | X |  | – | – | – | X | – | X | X |
|  | 6 | X | X | – |  | – | – | – | – | X | X | X | X |
|  | 24 | X | X | X |  | – | X | – | – | X | – | X | X |
| SBP | 3 | – | X | X | X |  | – | – | – | – | – | X | X |
|  | 6 | X | X | X |  | – | – | – | X | – | – | X | X |
|  | 24 | X | X | X |  | – | – | – | – | X | X | X | X |
| DBP | 3 | – | X | – | – |  | – | – | – | – | – | – | X |
|  | 6 | X | X | – |  | – | – | – | X | – | – | – | X |
|  | 24 | X | X | – |  | – | – | – | – | X | X | – | X |
| SVR | 3 | – | X | X | X |  | – | – | X | X | – | – | X |
|  | 6 | X | X | X |  | – | – | – | X | X | X | X | X |
|  | 24 | X | X | X |  | – | – | – | X | X | – | – | X |

CI, cardiac index; CIN, cinaciguat; DBP, diastolic blood pressure; LEV, levosimendan; LEVp, levosimendan pilot study; NES, nesiritide; NESp, nesiritide pilot study; **PAWP**, pulmonary **arterial** wedge pressure; RAP, right atrial pressure; SBP, systolic blood pressure; SER, serelaxin; SVR, systemic vascular resistance; TEZhd, tezosentan high dose; TEZld, tezosentan low dose; TEZp, tezosentan pilot study; ULA, ularitide; ULAp, ularitide pilot study.

**b)**

| **Parameter** | **Time (hours)** | **ULAp** | **ULA** | **NESp** | **NES ≤3 h** | **NES >3 h** | **LEVp** | **LEV** | **TEZp** | **TEZld** | **TEZhd** | **CIN** | **SER** |
| --- | --- | --- | --- | --- | --- | --- | --- | --- | --- | --- | --- | --- | --- |
| BNP | 3 | – | – | X | – |  | – | – | – | – | – | – | – |
|  | 6 | – | – | – |  | – | – | – | X | – | – | – | – |
|  | 24 | – | – | – |  | – | – | – | X | – | – | X | – |
| NT-proBNP | 6 | X | X | – |  | – | – | – | – | – | – | – | – |
|  | 24 | X | X | – |  | – | – | – | – | – | – | – | – |
| Creatinine | 24 | X | X | – |  | – | – | X | – | – | X | – | – |

BNP, brain natriuretic peptide; CIN, cinaciguat; LEV, levosimendan; LEVp, levosimendan pilot study; NES, nesiritide; NESp, nesiritide pilot study; NT-proBNP, *N*-terminal pro-brain natriuretic peptide; SER, serelaxin; TEZhd, tezosentan high dose; TEZld, tezosentan low dose; TEZp, tezosentan pilot study; ULA, ularitide; ULAp, ularitide pilot study.

**c)**

| **Parameter** | **ULAp** | **ULA** | **NESp** | **NES ≤3 h** | **NES >3 h** | **LEVp** | **LEV** | **TEZp** | **TEZld** | **TEZhd** | **CIN** | **SER** |
| --- | --- | --- | --- | --- | --- | --- | --- | --- | --- | --- | --- | --- |
| Discontinuation | X | X | X | – | X | – | X | X | – | X | – | – |
| Discontinuation due to AE | X | X | X | – | X | – | X | X | – | X | – | – |
| AE | X | X | – | – | X | – | X | X | – | X | X | – |
| Serious AE | X | X | – | – | – | – | X | – | – | – | X | – |
| Mortality (at 30 days) | X | X | – | – | – | – | X | X | – | X | X | X |

AE, adverse event; CIN, cinaciguat; LEV, levosimendan; LEVp, levosimendan pilot study; NES, nesiritide; NES, nesiritide pilot study; SER, serelaxin; TEZhd, tezosentan high dose; TEZld, tezosentan low dose; TEZ, tezosentan pilot study; ULA, ularitide; ULAp, ularitide pilot study.
