# Supplementary material for: Randomized double‐blind clinical studies of ularitide and other vasoactive substances in acute decompensated heart failure: a systematic review and meta‐analysis
Source: ESC Heart Fail. 2018 Sep 24;5(6):1023–34. doi: 10.1002/ehf2.12349 (PMC6300812; doi:10.1002/ehf2.12349)
Supplement: Supplementary file 3 — Table S3. Number of patients in the selected dose groups of the investigational medical product (IMP) and the comparator product (Comp.) stratified into main and pilot studies. [file EHF2-5-1023-s003.docx]

**Table S3. Number of patients in the selected dose groups of the investigational medical product (IMP) and the comparator product (Comp.) stratified into main and pilot studies**

| **Stratum** | **Comp.** | **Studies** | **No. of patients** | |
| --- | --- | --- | --- | --- |
|  |  |  | **IMP** | **Comp.** |
| Main | Placebo | ULA | 53 | 53 |
|  |  | NES ≤3 h; TEZld; TEZhd; CIN; SER | 388 | 285 |
|  | Active | NES >3 h; LEV | 249 | 185 |
| Pilot | Placebo | ULAp | 6 | 6 |
|  |  | NESp; LEVp; TEZp | 72 | 76 |
| Total |  | Including NES ≤3 h | 622 | 520 |
|  |  | Including NES >3 h | 644 | 543 |

CIN, cinaciguat; LEV, levosimendan; LEVp, levosimendan pilot study; NES, nesiritide; NESp, nesiritide pilot studySER, serelaxin; TEZhd, tezosentan high dose; TEZld, tezosentan low dose; TEZ, tezosentan pilot study; ULA, ularitide; ULAp, ularitide pilot study.
