# Supplementary material for: Randomized double‐blind clinical studies of ularitide and other vasoactive substances in acute decompensated heart failure: a systematic review and meta‐analysis
Source: ESC Heart Fail. 2018 Sep 24;5(6):1023–34. doi: 10.1002/ehf2.12349 (PMC6300812; doi:10.1002/ehf2.12349)
Supplement: Supplementary file 4 — Table S4. Heterogeneity in the OTHER studies: a) Controlled main studies; b) Placebo‐controlled main studies; c) All controlled studies. [file EHF2-5-1023-s004.docx]

**Table S4. Heterogeneities in the OTHER studies: a) Controlled main studies; b) Placebo-controlled main studies; c) All controlled studies**

**a)**

| **Parameter^a^** | **I^2^ (*P*-value)** | | |
| --- | --- | --- | --- |
|  | **3 hours of treatment** | **6 hours of treatment** | **24 hours of treatment** |
| **PAWP** | 52.0% (0.0800) | 52.9% (0.0595) | 6.6% (0.3691) |

^a^ No further target parameters available in active-comparator studies, therefore, the presentation was completed in b).

**PAWP**, pulmonary **arterial** wedge pressure.

**b)**

| **Parameter** | **I^2^ (*P*-value)** | | |
| --- | --- | --- | --- |
|  | **3 hours of treatment** | **6 hours of treatment** | **24 hours of treatment** |
| **PAWP** | 0.0% (0.6924) | 0.0% (0.5968) | 27.6% (0.2514) |
| CI | 58.4% (0.0476) | 80.0% (0.0018) | 84.4% (0.0002) |
| RAP | 47.5% (0.1265) | 0.0% (0.8011) | 0.0% (0.8133) |
| SBP | 0.0% (0.6159) | 64.2% (0.0946) | 0.0% (0.4599) |
| DBP | 0.0% (1.0000) | 0.0% (1.0000) | 0.0% (0.6786) |
| SVR | 0.0% (0.8485) | 85.8% (<0.0001) | 0.0% (0.9994) |
| Discontinuation | 0.0% (1.0000) | | |
| Discontinuation due to AE | 0.0% (1.0000) | | |
| AE | 0.0% (0.5673) | | |
| Serious AE | 0.0% (1.0000) | | |

AE, adverse event; CI, cardiac index; DBP, diastolic blood pressure; **PAWP, pulmonary arterial wedge pressure**; RAP, right atrial pressure; SBP, systolic blood pressure; SVR, systemic vascular resistance.

**c)**

| **Parameter** | **I^2^ (*P*-value)** | | |
| --- | --- | --- | --- |
|  | **3 hours of treatment** | **6 hours of treatment** | **24 hours of treatment** |
| **PAWP** | 47.0% (0.0671) | 64.4% (0.0041) | 40.4% (0.1094) |
| CI | 47.6% (0.0756) | 80.4% (0.0001) | 75.8% (0.0009) |
| RAP | 40.9% (0.1486) | 0.0% (0.8011) | 0.0% (0.9146) |
| SBP | 9.7% (0.3445) | 36.2% (0.1949) | 0.0% (0.5188) |
| DBP | 0.0% (1.0000) | 0.0% (0.9539) | 0.0% (0.6786) |
| SVR | 0.0% (0.8906) | 80.0% (0.0001) | 0.0% (0.9828) |
| BNP/NT-proBNP |  | 0.0% (1.0000) | 0.0% (0.5140) |
| Serum creatinine |  |  | 95.2% (<0.0001) |

BNP, brain natriuretic peptide; CI, cardiac index; DBP, diastolic blood pressure; NT-proBNP, *N*-terminal pro-brain natriuretic peptide; **PAWP, pulmonary arterial wedge pressure**; RAP, right atrial pressure; SBP, systolic blood pressure; SVR, systemic vascular resistance.
