# Supplementary material for: Randomized double‐blind clinical studies of ularitide and other vasoactive substances in acute decompensated heart failure: a systematic review and meta‐analysis
Source: ESC Heart Fail. 2018 Sep 24;5(6):1023–34. doi: 10.1002/ehf2.12349 (PMC6300812; doi:10.1002/ehf2.12349)
Supplement: Supplementary file 5 — Table S5. Hedges' g scores (95% CIs) of haemodynamic parameters for ularitide vs. placebo and the synthesis of all other study treatments vs. placebo (random‐effects model; placebo‐controlled main studies). [file EHF2-5-1023-s005.docx]

**Table S5. Hedges’ g scores (95% CIs) of haemodynamic parameters for ularitide vs. placebo and the synthesis of all other study treatments vs. placebo (random-effects model; placebo-controlled main studies)**

| **Parameter** | **Hedges’ g [95% CI]** | | **Indirect comparison of ularitide with other treatments** | |
| --- | --- | --- | --- | --- |
|  | **Random-effects model for ularitide vs. placebo** | **Random-effects model for other study treatments vs. placebo** | **Difference ularitide – others** | ***P*-value** |
| 3 hours |  |  |  |  |
| **PAWP** | –0.816 [–1.215, –0.417]^d^ | –0.538 [–0.728, –0.348]^d^ | –0.278 | 0.2174 |
| Cardiac index | 0.583 [0.193, 0.972]^b^ | 0.351 [0.099, 0.603]^b^ | 0.231 | 0.3284 |
| RAP | –0.469 [–0.856, –0.082]^a^ | –0.454 [–0.723, –0.186]^c^ | –0.015 | 0.9518 |
| SBP | –0.207 [–0.590, 0.175] | –0.339 [–0.546, –0.133]^b^ | 0.132 | 0.5512 |
| DBP | –0.564 [–0.953, –0.175]^b^ | –0.420 [–0.913, 0.073] | –0.144 | 0.6539 |
| SVR | –0.612 [–1.010, –0.213]^b^ | –0.263 [–0.485, –0.040]^a^ | –0.349 | 0.1339 |
| 6 hours |  |  |  |  |
| **PAWP** | –0.979 [–1.383, –0.575]^d^ | –0.649 [–0.842, –0.457]^d^ | –0.330 | 0.1483 |
| Cardiac index | 0.530 [0.142, 0.919]^b^ | 0.452 [0.019, 0.885]^a^ | 0.078 | 0.7914 |
| RAP | –0.797 [–1.193, –0.400]^d^ | –0.304 [–0.490, –0.118]^b^ | –0.493 | 0.0274 |
| SBP | –0.424 [–0.810, –0.038]^a^ | –0.545 [–1.048, –0.042]^a^ | 0.121 | 0.7075 |
| DBP | –0.586 [–0.976, –0.196]^b^ | –0.312 [–0.802, 0.179] | –0.274 | 0.3910 |
| SVR | –0.583 [–0.980, –0.185]^b^ | –0.705 [–1.231, –0.179]^b^ | 0.122 | 0.7161 |
| 24 hours |  |  |  |  |
| **PAWP** | –0.610 [–1.001, –0.220]^b^ | –0.304 [–0.599, –0.010]^a^ | –0.306 | 0.2206 |
| Cardiac index | 0.330 [–0.054, 0.715] | 0.430 [–0.074, 0.934] | –0.100 | 0.7577 |
| RAP | –0.378 [–0.763, 0.007] | –0.147 [–0.396, 0.102] | –0.231 | 0.3230 |
| SBP | –0.432 [–0.818, –0.046]^a^ | –0.414 [–0.599, –0.230]^d^ | –0.017 | 0.9367 |
| DBP | –0.607 [–0.997, –0.216]^b^ | –0.447 [–0.664, –0.230]^d^ | –0.160 | 0.4836 |
| SVR | –0.633 [–1.032, –0.235]^b^ | –0.271 [–0.595, 0.053] | –0.362 | 0.1668 |

CI, confidence interval; DBP, diastolic blood pressure; **PAWP, pulmonary arterial wedge pressure**; RAP, right atrial pressure; SBP, systolic blood pressure; SVR, systemic vascular resistance.

^a^ *P* < 0.05; ^b^ *P* < 0.01; ^c^ *P* < 0.001; ^d^ *P* < 0.0001 vs. placebo.
