# Supplementary material for: Randomized double‐blind clinical studies of ularitide and other vasoactive substances in acute decompensated heart failure: a systematic review and meta‐analysis
Source: ESC Heart Fail. 2018 Sep 24;5(6):1023–34. doi: 10.1002/ehf2.12349 (PMC6300812; doi:10.1002/ehf2.12349)
Supplement: Supplementary file 6 — Table S6. Hedges' g scores (95% CIs) of other endpoints for the synthesis of ularitide vs. placebo and the synthesis of all other study treatments vs. comparator (random‐effects model; placebo‐controlled main studies). [file EHF2-5-1023-s006.docx]

**Table S6**. **Hedges’ g scores (95% CIs) of other endpoints for the synthesis of ularitide vs. placebo and the synthesis of all other study treatments vs. comparator (random-effects model; placebo-controlled main studies)**

| **Parameter** | **Hedges’ g [95% CI]** | | **Indirect comparison of ularitide with other treatments** | |
| --- | --- | --- | --- | --- |
|  | **Random-effects model for ularitide vs. placebo** | **Random-effects model for other study treatments vs. comparator** | **Difference ularitide – other treatments** | ***P*-value** |
| 6 hours |  |  |  |  |
| BNP/ NT-proBNP | –0.085 [–0.452, 0.281] | –0.431 [–1.199, 0.337] | 0.345 | 0.4264 |
| 24 hours |  |  |  |  |
| BNP/ NT-proBNP | –0.578 [–0.952, –0.204] ^a^ | –0.531 [–0.882, –0.181] ^a^ | –0.046 | 0.8589 |
| Serum creatinine | –0.153 [–0.525, 0.220] | –0.030 [–0.975, 0.915] | –0.123 | 0.8127 |

CI, confidence interval; NT-proBNP, *N*-terminal pro-brain natriuretic peptide.

^a^ *P* < 0.01.
