# Supplementary material for: Randomized double‐blind clinical studies of ularitide and other vasoactive substances in acute decompensated heart failure: a systematic review and meta‐analysis
Source: ESC Heart Fail. 2018 Sep 24;5(6):1023–34. doi: 10.1002/ehf2.12349 (PMC6300812; doi:10.1002/ehf2.12349)
Supplement: Supplementary file 7 — Table S7. Risk ratios (95% CIs) of safety results for ularitide vs. placebo and the synthesis of all other treatments vs. placebo (random‐effects model; placebo‐controlled main studies). [file EHF2-5-1023-s007.docx]

**Table S7**. **Risk ratios (95% CIs) of safety results for ularitide vs. placebo and the synthesis of all other treatments vs. placebo (random-effects model; placebo-controlled main studies)**

| **Parameter** | **Risk ratio [95% CI]** | | **Indirect comparison of ularitide with other treatments** | |
| --- | --- | --- | --- | --- |
|  | **Random-effects model for ularitide vs. placebo** | **Random-effects model for other study treatments vs. comparator** | **Risk ratio other/ ularitide** | ***P*-value** |
| Discontinuation | 3.000 [0.125, 72.018] | 3.133 [0.649, 15.121] | 1.044 | 0.9808 |
| Discontinuation due to AE | 3.000 [0.125, 72.018] | 3.133 [0.649, 15.121] | 1.044 | 0.9808 |
| AE | 1.182 [0.583, 2.396] | 1.460 [1.201, 1.776] | 1.236 | 0.5716 |
| Serious AE | 1.000 [0.146, 6.839] | 4.732 [0.617, 36.318] | 4.732 | 0.2769 |

AE, adverse event; CI, confidence interval.
