# Supplementary material for: Randomized double‐blind clinical studies of ularitide and other vasoactive substances in acute decompensated heart failure: a systematic review and meta‐analysis
Source: ESC Heart Fail. 2018 Sep 24;5(6):1023–34. doi: 10.1002/ehf2.12349 (PMC6300812; doi:10.1002/ehf2.12349)
Supplement: Supplementary file 8 — Figure S1. Mean changes in: a) PAWP; b) cardiac index at 3, 6, and 24 hours with active compounds. Bars are the mean with standard error. CIN, cinaciguat; LEV, levosimendan; PAWP, pulmonary arterial wedge pressure; NES, nesiritide; NIT, nitroglycerin; SER, serelaxin; TEZhd, tezosentan high dose; TEZld, tezosentan low dose; ULA, ularitide. Figure S2. Hedges' g scores (95% CIs) of changes from baseline to: a) 3 hours; b) 24 hours in pulmonary arterial wedge pressure in the ularitide study SIRIUS II (ULA)1 and the synthesis of all other controlled main studies (OTH). CI, confidence interval; CIN, cinaciguat; LEV, levosimendan; LL, lower limit; NES, nesiritide; NIT, nitroglycerin; SER, serelaxin; TEZhd, tezosentan high dose; TEZld, tezosentan low dose; UL, upper limit; ULA, ularitide. Figure S3. Risk ratios (RRs) (95% CIs) of 30‐day mortality rates in the ularitide study SIRIUS II (ULA)1 and the synthesis of the placebo‐controlled main studies. CI, confidence interval; CIN, cinaciguat; LL, lower limit; SER, serelaxin; TEZhd, tezosentan high dose; UL, upper limit; ULA, ularitide. Figure S4. Sensitivity analysis. Hedges' g scores (95% CIs) of changes from baseline to: a) 3 hours; b) 6 hours; c) 24 hours in pulmonary arterial wedge pressure in the ularitide study SIRIUS II (ULA)1 and the synthesis of all other placebo‐controlled main studies (OTH). CI, confidence interval; CIN, cinaciguat; LEV, levosimendan; LL, lower limit; NES, nesiritide; PAWP, pulmonary arterial wedge pressure; SER, serelaxin; TEZhd, tezosentan high dose; TEZld, tezosentan low dose; UL, upper limit; ULA, ularitide. Figure S5. Sensitivity analysis: Hedges' g scores (95% CIs) of changes from baseline to: a) 3 hours; b) 6 hours; c) 24 hours in pulmonary arterial wedge pressure in the ularitide studies SIRIUS I8 and II (ULAp, ULA)1 and the synthesis of all other controlled pilot and main studies (OTH). CI, confidence interval; CIN, cinaciguat; LEV, levosimendan; LL, lower limit; NES, nesiritide; PAWP, pulm [file EHF2-5-1023-s008.docx]

### Appendix

#### Statistical methods

Synthesis of effect sizes across studies was performed using a fixed-effect model and a random-effects model. The fixed-effect model assumes that there is one true effect that underlies all studies in the analysis, and that all differences in observed effects are the result of sampling error. The random-effects model assumes that the true effect can vary from study to study; effect sizes in the studies that were performed are assumed to represent a random sample of these effect sizes.^1^ The synthesized effect sizes are weighted means of the individual results. In the fixed-effect model, the weights depend only on the variances of the respective effect measures, whereas the weights in the random-effects model also depend on between-study variance. Primary analyses were based on the random-effects model.

The risk of bias in the individual studies was assessed using the Cochrane risk of bias tool.^2^ The risk of bias across studies was assessed by means of a so-called I^2^ value, which is defined as between-study variance as a proportion of total variance, expressed as a percentage and calculated in the fixed-effect model.

#### Sensitivity meta-analyses of primary and secondary haemodynamic endpoints

Sensitivity meta-analyses were performed in placebo-controlled main studies and in all controlled studies (including active comparators and pilot studies). In the main placebo-controlled studies, ularitide was superior to all other treatments in terms of **PAWP** at 3, 6, and 24 hours (Hedges’ g, –0.816 vs. –0.538, –0.979 vs. –0.649, and –0.610 vs. –0.304, respectively), although this was not statistically significant (*Figure S4*). Results were similar for ularitide vs. other treatments in all controlled pilot and main studies (–0.816 vs. –0.467,–0.923 vs. –0.560, and –0.426 vs. –0.447, respectively) (*Figure S5*). At 3 and 6 hours, the treatment differences were –0.349 (95% CI: –0.798, 0.099; *P* = 0.1270) and –0.364 (95% CI: –0.803, 0.076; *P* = 0.1050), respectively.

#### References

1. Borenstein M, Hedges LV, Higgins JPT, Rothstein HR. *Introduction to Meta-analysis.* Hoboken, NJ: John Wiley & Sons; 2009.

2. Higgins JPT, Green S. *Cochrane Handbook for Systematic Reviews of Interventions Version 5.1.0*. London: The Cochrane Collaboration; 2011.

#### Use of digitization for data extraction

The following figures and tables contain data extracted from figures with WebPlotDigitiser versions 3.8 and 3.9, valid between May 2015 and May 2016 (web-based software developed by Ankit Rohatgi, Austin, Texas, USA and freely available at http://arohatgi.info/WebPlotDigitizer/). At least data of one parameter, one study or one time point were digitized):

- *Figure 3* Mean 24-hour changes in **PAWP** and cardiac index. Time course of mean changes from baseline to 24 hours in (a) **PAWP** and (b) cardiac index.
- *Figure 4* **PAWP** and RAP effect sizes for ularitide vs. other controlled main studies.
- Hedges’ g scores (95% CIs) of changes from baseline to 6 hours in **PAWP** (a) and in RAP (b) in the ularitide study SIRIUS II (ULA)^9^ and the synthesis of all other controlled main studies (OTH).
- *Table S4* Heterogeneities in the OTHER Studies: a) Controlled main studies; b) Placebo-controlled main studies; c) All controlled studies
- *Table S5* Hedges’ g scores (95% CIs) of haemodynamic parameters for ularitide vs. placebo and the synthesis of all other study treatments vs. placebo (random-effects model; placebo-controlled main studies)
- *Figure 2* Mean changes in **PAWP** at 6 hours with ularitide vs. placebo and other agents vs. comparator (Comp.; other active treatment or placebo). Bars are the mean with standard error.
- *Figure S1* Mean changes in: a) **PAWP**; b) CI at 3, 6, and 24 hours with active compounds. Bars are the mean with standard error.
- *Figure S2* Hedges’ g scores (95% CIs) of changes from Baseline to: a) 3 hours; b) 24 hours in pulmonary **arterial** wedge pressure in the ularitide study SIRIUS II (ULA)^9^ and the synthesis of all other controlled main studies (OTH). **[Note: not for a) 3 hours]**
- *Figure S4* Sensitivity analysis. Hedges’ g scores (95% CIs) of changes from baseline to: a) 3 hours; b) 6 hours; c) 24 hours in pulmonary **arterial** wedge pressure in the ularitide study SIRIUS II (ULA)^9^ and the synthesis of all other placebo-controlled main studies (OTH). **[Note: not for a) 3 hours]**
- *Figure S5* Sensitivity analysis: Hedges’ g scores (95% CIs) of changes from baseline to: a) 3 hours; b) 6 hours; c) 24 hours in pulmonary **arterial** wedge pressure in the ularitide studies SIRIUS I^8^ and II (ULAp, ULA)^9^ and the synthesis of all other controlled pilot and main studies (OTH).

#### Supplementary figure legends

**Figure S1** Mean changes in: a) **PAWP**; b) cardiac index at 3, 6, and 24 hours with active compounds. Bars are the mean with standard error.

CIN, cinaciguat; LEV, levosimendan; **PAWP, pulmonary arterial wedge pressure**; NES, nesiritide; NIT, nitroglycerin; SER, serelaxin; TEZhd, tezosentan high dose; TEZld, tezosentan low dose; ULA, ularitide.

**Figure S2** Hedges’ g scores (95% CIs) of changes from Baseline to: a) 3 hours; b) 24 hours in pulmonary **arterial** wedge pressure in the ularitide study SIRIUS II (ULA)^9^ and the synthesis of all other controlled main studies (OTH).

CI, confidence interval; CIN, cinaciguat; LEV, levosimendan; LL, lower limit; NES, nesiritide; NIT, nitroglycerin; SER, serelaxin; TEZhd, tezosentan high dose; TEZld, tezosentan low dose; UL, upper limit; ULA, ularitide.

**Figure S3** Risk ratios (RRs) (95% CIs) of 30-day mortality rates in the ularitide study SIRIUS II (ULA)^9^ and the synthesis of the placebo-controlled main studies.

CI, confidence interval; CIN, cinaciguat; LL, lower limit; SER, serelaxin; TEZhd, tezosentan high dose; UL, upper limit; ULA, ularitide.

**Figure S4** Sensitivity analysis. Hedges’ g scores (95% CIs) of changes from baseline to: a) 3 hours; b) 6 hours; c) 24 hours in **pulmonary arterial wedge pressure** in the ularitide study SIRIUS II (ULA)^9^ and the synthesis of all other placebo-controlled main studies (OTH).

CI, confidence interval; CIN, cinaciguat; LEV, levosimendan; LL, lower limit; NES, nesiritide; **PAWP**, pulmonary **arterial** wedge pressure; SER, serelaxin; TEZhd, tezosentan high dose; TEZld, tezosentan low dose; UL, upper limit; ULA, ularitide.

**Figure S5** Sensitivity analysis: Hedges’ g scores (95% CIs) of changes from baseline to: a) 3 hours; b) 6 hours; c) 24 hours in **pulmonary arterial wedge pressure** in the ularitide studies SIRIUS I^8^ and II (ULAp, ULA)^9^ and the synthesis of all other controlled pilot and main studies (OTH).

CI, confidence interval; CIN, cinaciguat; LEV, levosimendan; LL, lower limit; NES, nesiritide; **PAWP**, pulmonary **arterial** wedge pressure; SER, serelaxin; TEZhd, tezosentan high dose; TEZld, tezosentan low dose; UL, upper limit; ULA, ularitide.
