# Supplementary figures and images for: Randomized double‐blind clinical studies of ularitide and other vasoactive substances in acute decompensated heart failure: a systematic review and meta‐analysis
Source: ESC Heart Fail. 2018 Sep 24;5(6):1023–34. doi: 10.1002/ehf2.12349 (PMC6300812; doi:10.1002/ehf2.12349)

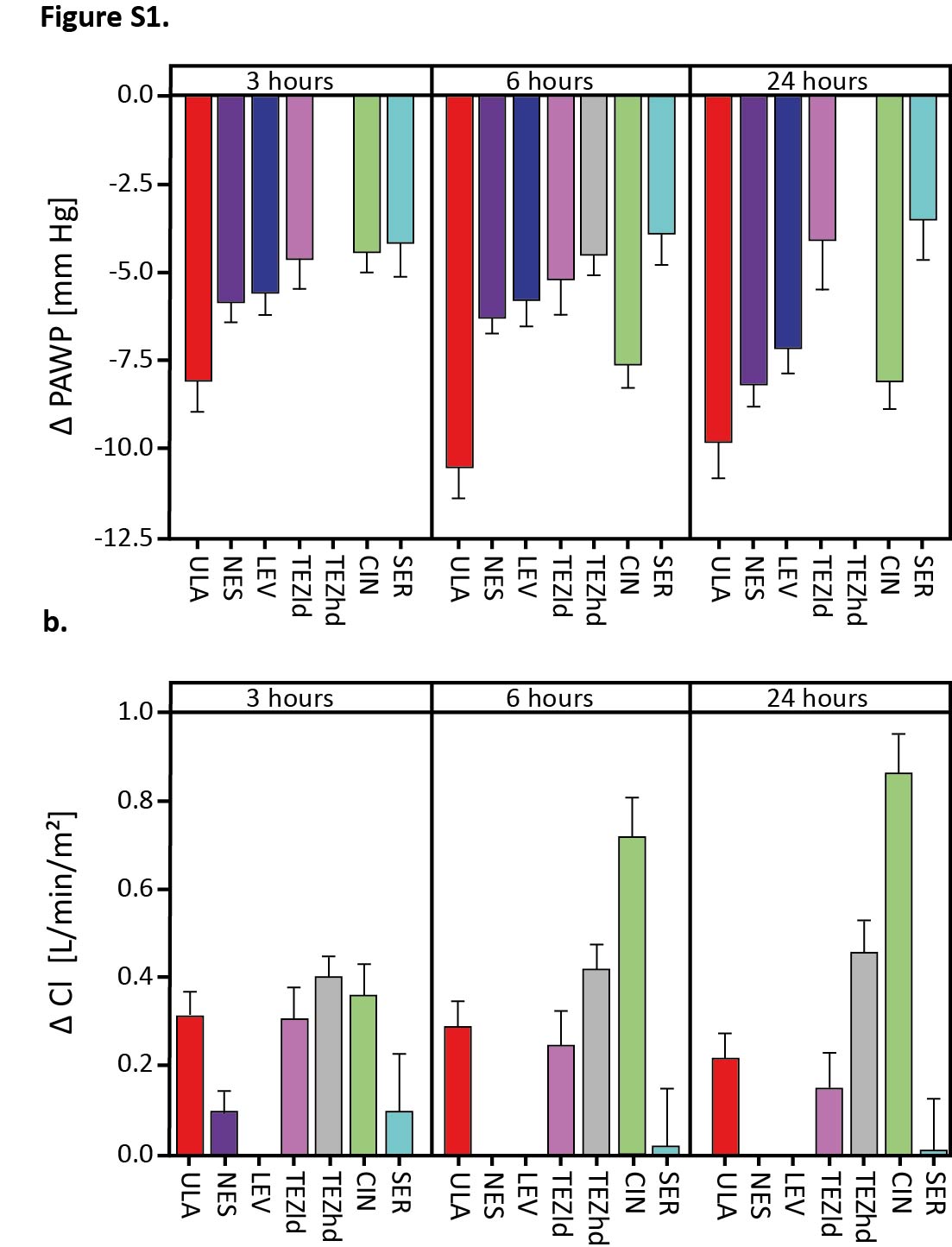

Supplement: Supplementary file 9 — Supporting info item [file EHF2-5-1023-s009.jpg]

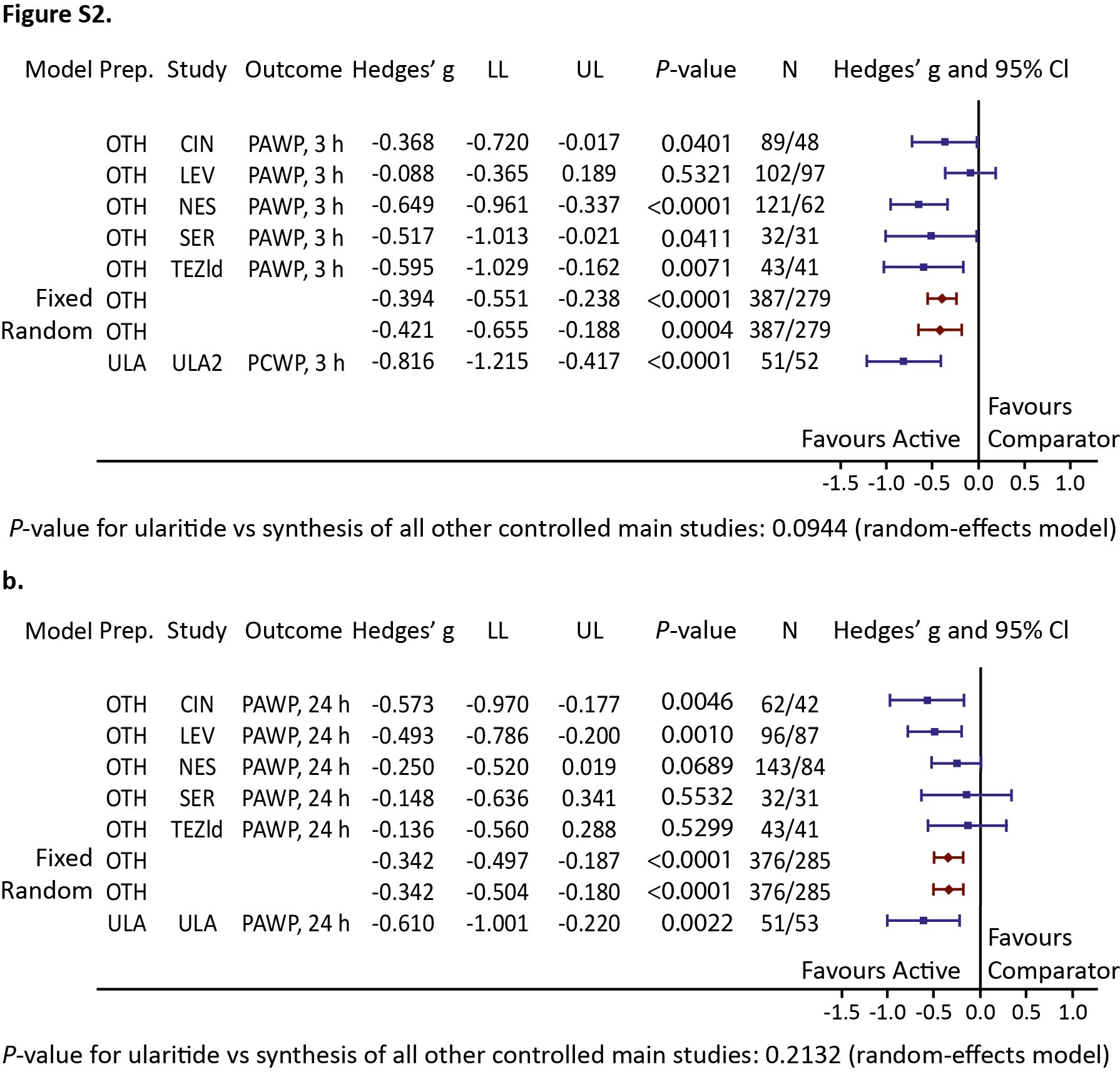

Supplement: Supplementary file 10 — Supporting info item [file EHF2-5-1023-s010.jpg]

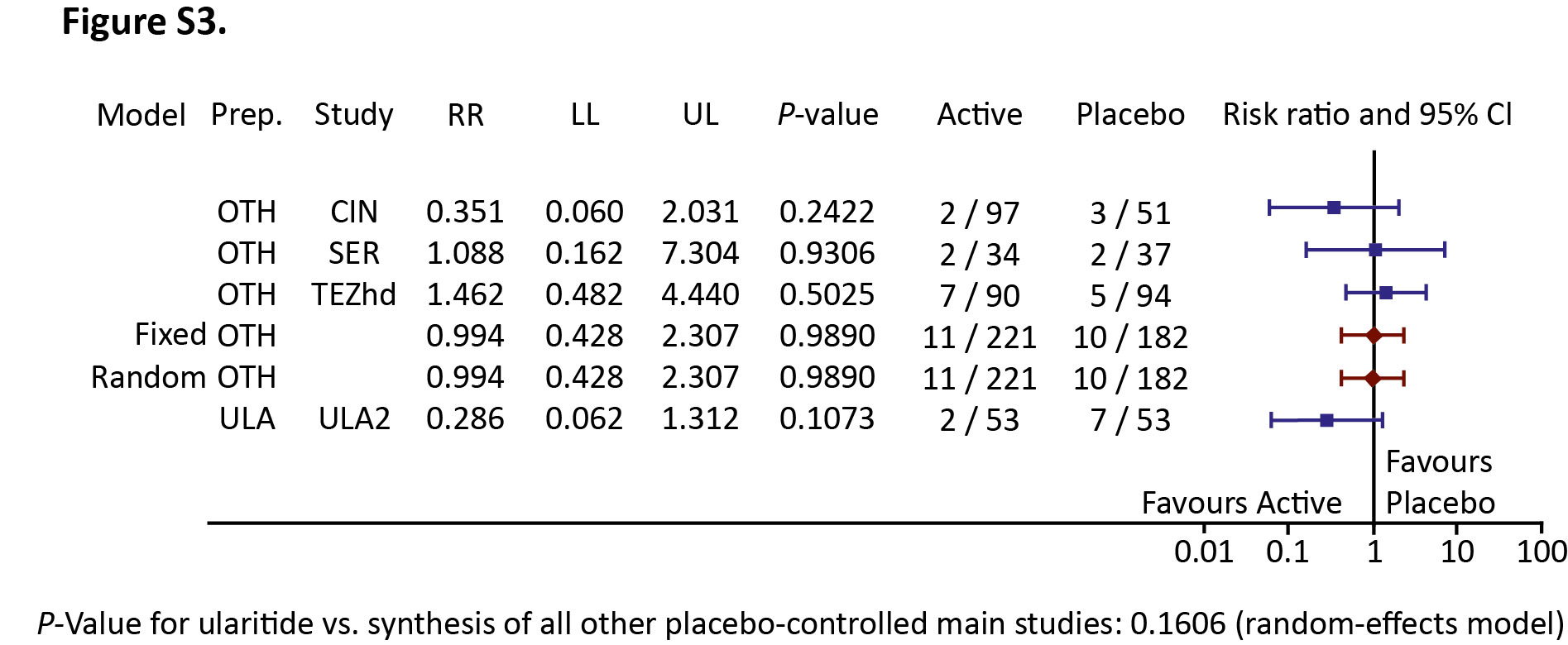

Supplement: Supplementary file 11 — Supporting info item [file EHF2-5-1023-s011.jpg]

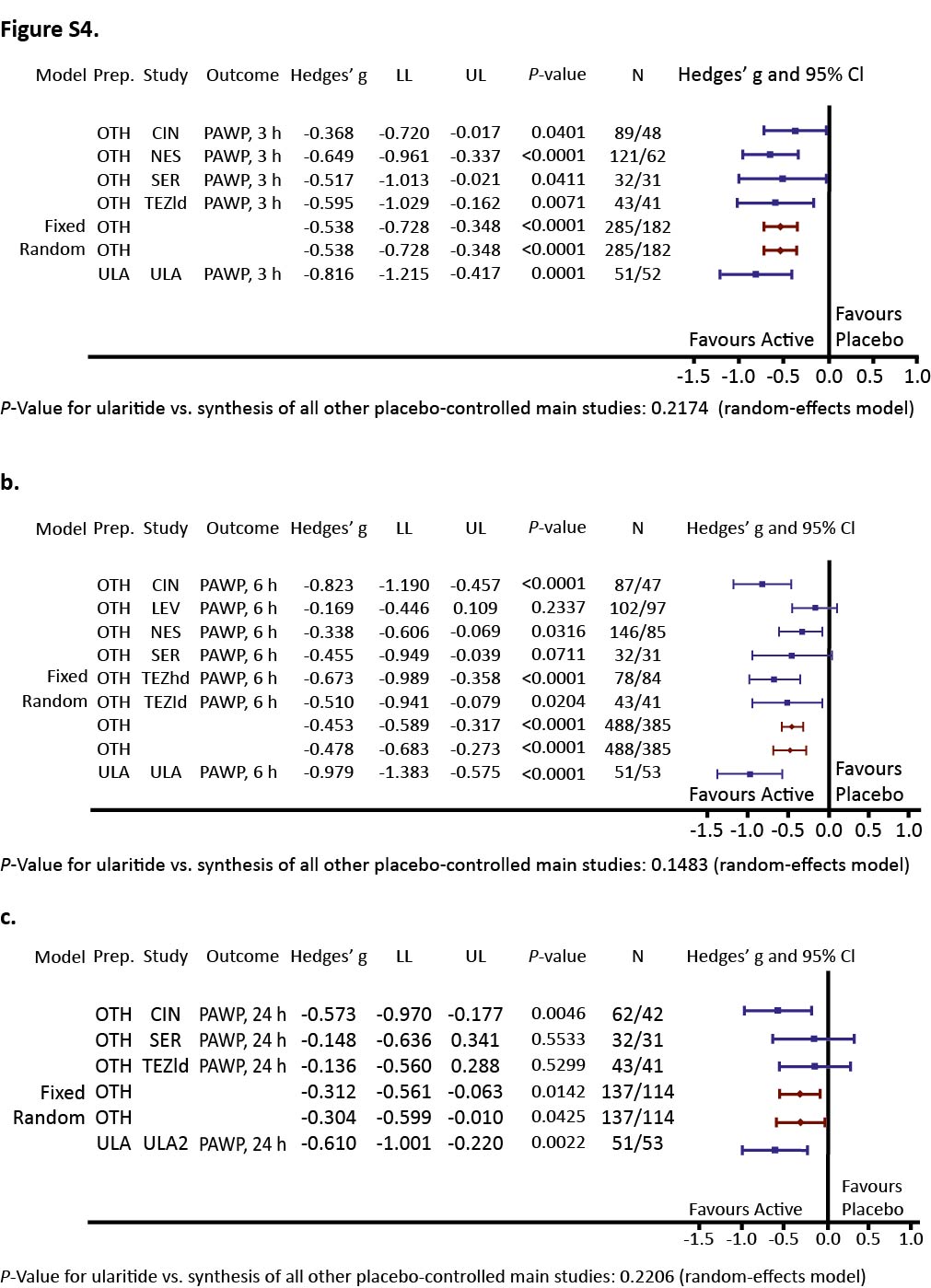

Supplement: Supplementary file 12 — Supporting info item [file EHF2-5-1023-s012.jpg]

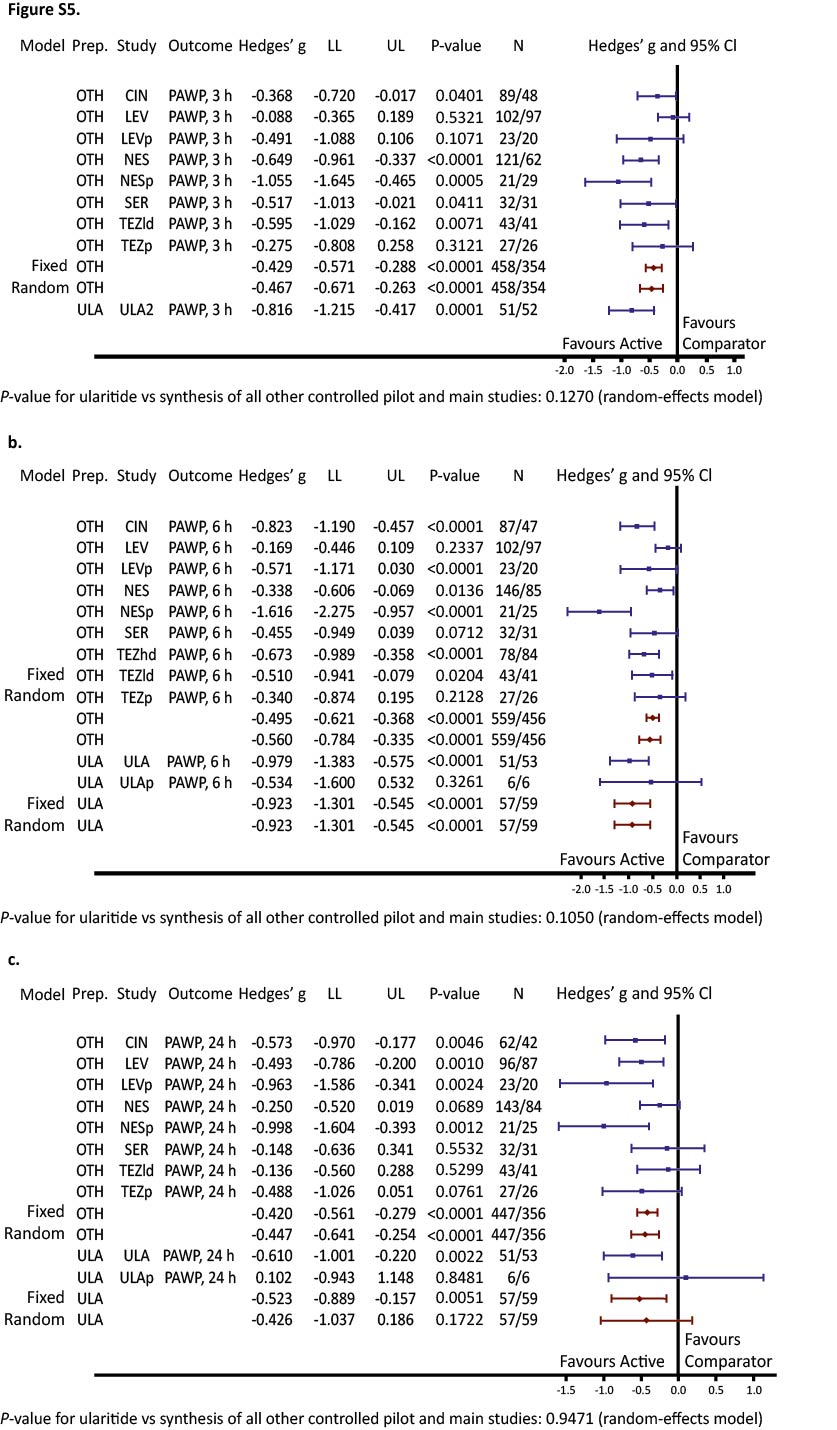

Supplement: Supplementary file 13 — Supporting info item [file EHF2-5-1023-s013.jpg]
